# KHSRP loss increases neuronal growth and synaptic transmission and alters memory consolidation through RNA stabilization

**DOI:** 10.1038/s42003-022-03594-4

**Published:** 2022-07-07

**Authors:** Sarah L. Olguin, Priyanka Patel, Courtney N. Buchanan, Michela Dell’Orco, Amy S. Gardiner, Robert Cole, Lauren S. Vaughn, Anitha Sundararajan, Joann Mudge, Andrea M. Allan, Pavel Ortinski, Jonathan L. Brigman, Jeffery L. Twiss, Nora I. Perrone-Bizzozero

**Affiliations:** 1 Department of Neurosciences, University of New Mexico School of Medicine, Albuquerque, NM 87131 USA; 2 Department of Biological Sciences, University of South Carolina, Columbia, SC 29208 USA; 3 Department of Neuroscience, University of Kentucky, Lexington, KY 40536 USA; 4 National Center for Genome Resources, Santa Fe, NM 87505 USA; 5 Carolina Autism and Neurodevelopment Center, University of South Carolina, Columbia, SC 29208 USA

**Keywords:** Molecular neuroscience, Cellular neuroscience

## Abstract

The KH-type splicing regulatory protein (KHSRP) is an RNA-binding protein linked to decay of mRNAs with AU-rich elements. KHSRP was previously shown to destabilize *Gap43* mRNA and decrease neurite growth in cultured embryonic neurons. Here, we have tested functions of KHSRP in vivo. We find upregulation of 1460 mRNAs in neocortex of adult *Khsrp*^−/−^ mice, of which 527 bind to KHSRP with high specificity. These KHSRP targets are involved in pathways for neuronal morphology, axon guidance, neurotransmission and long-term memory. *Khsrp*^*−/−*^ mice show increased axon growth and dendritic spine density in vivo. Neuronal cultures from *Khsrp*^*−/−*^ mice show increased axon and dendrite growth and elevated KHSRP-target mRNAs, including subcellularly localized mRNAs. Furthermore, neuron-specific knockout of *Khsrp* confirms these are from neuron-intrinsic roles of KHSRP. Consistent with this, neurons in the hippocampus and infralimbic cortex of *Khsrp*^*−/−*^ mice show elevations in frequency of miniature excitatory postsynaptic currents. The *Khsrp*^−/−^ mice have deficits in trace conditioning and attention set-shifting tasks compared *Khsrp*^*+/+*^ mice, indicating impaired prefrontal- and hippocampal-dependent memory consolidation with loss of KHSRP. Overall, these results indicate that deletion of KHSRP impairs neuronal development resulting in alterations in neuronal morphology and function by changing post-transcriptional control of neuronal gene expression.

## Abstract (web summary)

KH-type splicing regulatory protein (KHSRP) regulates levels of several neuronal mRNAs linked to nervous system development and function.

## Introduction

Post-transcriptional regulation of gene expression plays a critical role in neuronal differentiation and function. Independent from transcription and translation, these mechanisms are especially important in control of specific sets of mRNAs that localize into dendrites and axons^1^. Stability of mRNAs is also critically important for the regulation of gene expression, as changes in mRNA decay rates can be rapid and precise. mRNA sequences (*cis*-elements) are bound by *trans*-acting factors like RNA-binding proteins (RBPs) and miRNAs to effect changes in mRNA decay rates^2^. Binding by RBPs can stabilize an mRNA by protecting it from nucleases, promote its translation by targeting the mRNA to polysomes, or promote its decay by targeting the bound mRNA to RNA degradation sites in the cell^3^.

The KH-type splicing regulatory protein (KHSRP; also known as KSRP, FUBP2, ZBP2, and MARTA1) is an RBP implicated in decay of AU-rich element (ARE)-containing mRNAs by targeting them to the cytoplasmic exosome complex for degradation^4,5^. KHSRP was independently discovered as a single-stranded DNA binding protein, termed Far Upstream Element (FUSE) binding protein 2 (FUBP2), and a KH-homology RBP that enhanced splicing of the neuron-specific *c-Src* N1 exon^6,7^. KHSRP’s function has been linked to disease conditions including viral infections, diabetes and cancer^8^, but KHSRP is also highly expressed in neural tissues, including in neurons, and it localizes into both axons and dendrites^9^. The KHSRP orthologue in rats, MARTA1, was reported to be required for transport of *Map2* mRNA into neuronal dendrites^10^. Similarly, the chicken orthologue, zip code binding protein 2 (ZBP2) is involved in targeting nuclear *Actb* mRNA to the cytoplasm^11^. We previously showed that KHSRP can destabilize the mRNA encoding growth-associated protein 43 (*Gap43*) and regulate neurite growth in cultured embryonic hippocampal neurons^12^. Despite that KHSRP has contributions to RNA splicing, transport and decay, KSHRP’s function in the brain has not been systematically defined.

Here, we have used a combination of molecular, cellular, electrophysiological and behavioral approaches to better understand the role of KHSRP in the brain. We find that KHSRP regulates multiple neuronal target mRNAs that are associated with nervous system development and function, including neuronal morphology, axonal growth, and synaptic functions. These gene expression findings are consistent with increased neurite growth upon loss of KHSRP, which is seen in neurons from both constitutive and neuron-specific conditional knockouts of the murine *Khsrp* gene. Loss of KHSRP increases spontaneous neurotransmission and disrupts hippocampal-dependent learning and prefrontal cortex function in the *Khsrp*^−/−^ mice. Our findings emphasize the critical role that post-transcriptional modulation of mRNA levels by KHSRP plays in brain development and function.

## Results

### Neuronal KHSRP-target mRNAs are up-regulated in *Khsrp*^−/−^ brain

**Fig. 1:**
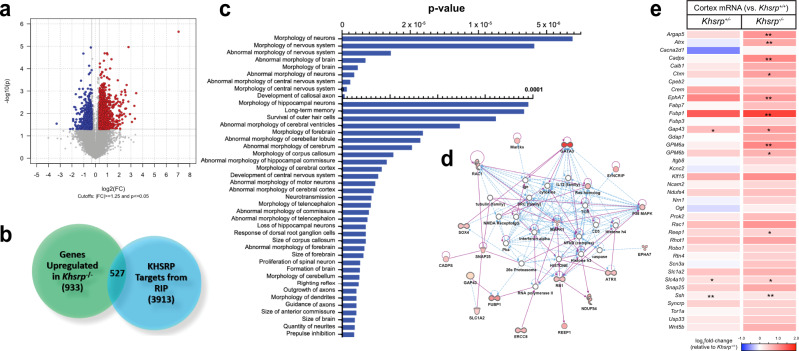
Increased levels of KHSRP-target mRNAs in the neocortex of KHSRP deficient mice. **a** Volcano Plot for differential expression of mRNAs in neocortex of *Khsrp*^*−/−*^ vs. *Khsrp*^*+/+*^ mice from microarray analyses. The vertical gray line corresponds to log_2_ fold-change (1.25 up or down), and the horizontal line represents a p-value of 0.05 that was used as a cut off for subsequent analyses. Also see Supplementary Data 1. **b** Venn diagram for overlap between genes significantly up-regulated in *Khsrp*^*−/−*^ vs. *Khsrp*^*+/+*^ and those identified as binding to KHSRP by RIP-seq (log_2_ fold-change > 1.4; *p* < 0.05). See Supplementary Data 2 for RNAs identified by KHSRP RIP-Seq and overlap with data from panel A and Supplementary Data 3 for ARE-containing KHSRP-target mRNAs). **c** IPA analyses of functional pathways regulated shapes by the 527 KHSRP-target mRNAs from b reveal top pathways related to neuronal morphology. P-values were calculated by comparing the frequency of the 527 genes in each pathway with the frequency expected by chance from all the genes in the genome using Fisher’s exact test at the right tail. **d** Top nervous system development and function network identified by IPA. Up-regulated genes are highlighted in red; color intensity is proportional to fold change increase in *Khsrp*^*−/−*^ cortex. **e** RTddPCR validation of alterations in levels of KHSRP-target mRNAs from d for *Khsrp*^*+/−*^ and *Khsrp*^*−/−*^ vs. *Khsrp*^*+/+*^ neocortex as a heat map based on log_2_ fold-changes for indicated mRNAs. See Supplementary Fig. 2c for validation of select KHSRP-target mRNAs in hippocampus and Supplementary Data 5 and 6 for mRNA copy number from these analyses (*N* ≥ 3 per condition; *p ≤ 0.05, **p ≤ 0.01, and ***p ≤ 0.005 by two-tailed Student’s *t-*test).

We have previously shown that KHSRP is expressed in cultured embryonic hippocampal neurons where it destabilizes *Gap-43* mRNA and attenuates neurite growth, while loss of KHSRP results in the opposite phenotype to increase both *Gap43* mRNA levels and neurite growth^12^. The affected neurites were inferred to be ‘axonal’ in those 5 day cultures of cortical neurons based on morphology. Here, we find that levels of KHSRP progressively increase as cultured neocortical neurons extend TuJ1-positive neurites that go on to polarize into axons and dendrites (Supplementary Fig. 1). Furthermore, KHSRP continues to be expressed in mature neocortical and hippocampal mouse neurons in vivo (Supplementary Fig. 2a, b). Although neuronal KHSRP protein shows expression from early development into adulthood, the function of this protein in neurons remains to be established in vivo. We used microarray analyses to systematically test for changes in mRNA levels in the neocortex of *Khsrp*^−/−^ vs. *Khsrp*^*+/+*^ adult mice. This identified 1460 mRNAs with significantly elevated levels and 2724 mRNAs that were significantly downregulated in neocortex upon loss of KHSRP expression (Fig. 1a, Supplementary Data 1). We next used RNA co-immunoprecipitation with KHSRP antibodies from neocortex of E18 wild type mice followed by next-generation sequencing (RIP-seq) to identify 4400 mRNAs bound to KHSRP primarily in neurons (Supplementary Data 2). Immunofluorescence and immunoblotting for KHSRP in wild type (*Khsrp*^*+/+*^) and *Khsrp* knockout (*Khsrp*^*−/−*^) mice shows specificity of the anti-KHSRP antibody (Supplementary Figs. 2a, b, d and 3). Integrating the RIP-Seq data and the set of up-regulated mRNAs from the microarrays enabled us to focus our subsequent analyses on a mRNA cohort that is potentially destabilized by direct interactions with KHSRP. From this, we identified 527 mRNAs that are both elevated in *Khsrp*^*−/-*^ vs. *Khsrp*^*+/+*^ neocortex and significantly enriched in KHSRP RIP-Seq from *Khsrp*^+/+^ vs. control RIP-Seq using *Khsrp*^−/−^ tissues; we refer to these as ‘KHSRP-target mRNAs’ (Fig. 1b; Supplementary Data 3). In contrast, only 144 downregulated transcripts were found to be enriched in the RIP (Supplementary Data 3). Among the up-regulated mRNAs, 444 have AU-rich elements (AREs) in their 3’ UTRs, suggesting that KHSRP can directly bind to and destabilize those transcripts (Supplementary Data 4). Analysis of the 527 KHSRP-target mRNAs using Ingenuity pathway analyses (IPA) revealed a significant enrichment of their encoded proteins in the control of neuronal morphology, axon development/growth, axonal guidance, long-term memory, neurotransmission, and other brain and neuron structure and function categories (Fig. 1c).

The top IPA nervous system development and function network derived from this list of KHSRP-target mRNAs showed 58 encoded proteins involved in neuronal morphology and axonal guidance with different extents of upregulation, including SNAP25, SYNCRIP, MARCKS, RAC1, and GAP43^13–16^ (Fig. 1d, Supplementary Data 3). Reverse-transcriptase-coupled droplet digital PCR (RTddPCR) with transcript specific primers validated the unbiased screens used in Fig. 1a, d, showing significantly increased levels for many KHSRP-target mRNAs in neocortex from adult *Khsrp*^*−/−*^ vs. *Khsrp*^*+/+*^ mice, with *Khsrp*^*+/−*^ samples often showing intermediate levels (Fig. 1e; Supplementary Data 5). A subset of these mRNAs also showed significantly increased levels in hippocampus dissected from the *Khsrp*^−/−^ and *Khsrp*^+/−^ vs. *Khsrp*^*+/+*^ mice (Supplementary Figs. 2c, 3, Supplementary Data 6). Several of the mRNAs validated for upregulation in the neocortex and hippocampus have been linked to neuronal differentiation and synaptic function beyond *Gap43* mRNA. For example, *Fubp1* mRNA encodes a member of the same FUSE protein binding family as KHSRP, and FUBP1 can promote terminal differentiation of neural progenitors^17^. *Snap25* encodes a member of the SNARE complex of the synaptic release machinery but has also been linked to axon growth and synapse development^18–20^. The Ephrin receptor EphA7 has been linked to neuronal differentiation, dendritic morphology, and LTP^21,22^. Finally, *Slc1a2* encodes the glial high affinity glutamate transporter EAAT2, but there is also evidence for *Slc1a2* expression by neurons^23^. Thus, in vivo loss of KHSRP protein expression alters levels of several different mRNAs whose protein products have clear potential to impact neuronal development and/or function. Immunoblots with protein lysates prepared from *Khsrp*^*+/+*^ and *Khsrp*^*−/−*^ mouse cortices confirmed an increase in SNAP25 and FUBP1 proteins upon loss of KHSRP (Supplementary Figs. 2d, 3).

### Loss of KHSRP increases axon and dendrite growth in vivo

**Fig. 2:**
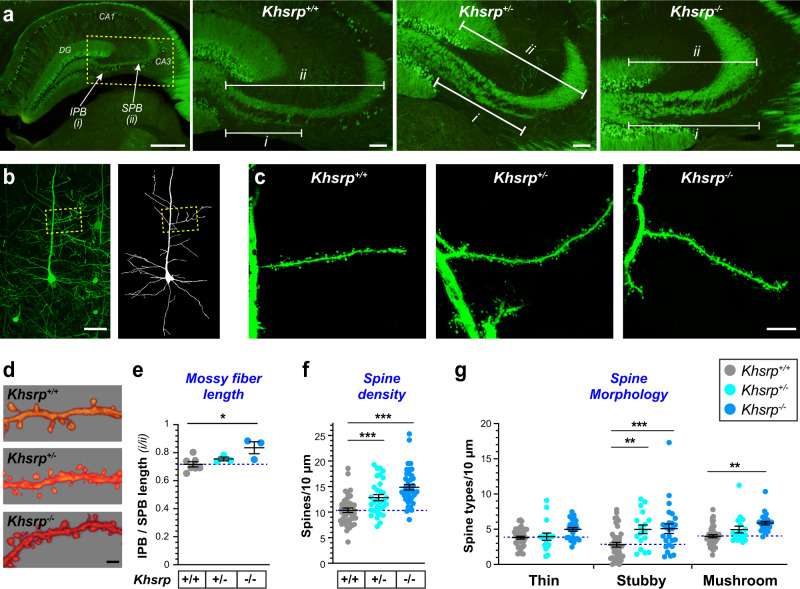
Increased axon and dendrite growth in the brains of KHSRP deficient mice. **a** Representative images for hippocampi of adult mice for Thy1-GFP crossed *with Khsrp*^*+/−*^ and *Khsrp*^*−/−*^ show altered mossy fiber outgrowth compared to *Khsrp*^*+/+*^. The low power image at left outlines area for three higher power images to the right. IPB is marked as ‘i’ and SPB as ‘ii’ in the three right panels (DG = dentate gyrus; CA1 and CA3 mark corresponding cornu Ammonis subfields). **b**–**d** Panel (**b**) shows a low magnification of confocal *XYZ* projection for layer 5 somatosensory cortex of Thy1-GFP × *Khsrp*^*−/−*^ mice; left panel shows fluorescent GFP signals and right panel shows Neurolucida tracing for a single neuron; boxed region shows approximate site for images of the secondary branches of apical dendrites shown in panels (**c**, **d**). Higher magnification images of apical dendrite branches for Thy1-GFP mice crossed with *Khsrp*^*+/−*^ and *Khsrp*^*−/−*^ show that loss of KHSRP increases dendritic spine numbers in panels (**c**) and (**d**). **e**–**g** Mean IPB/SPB length (i/ii) (**e**) is significantly increased in *Khsrp*^*−/−*^ compared to *Khsrp*^*+/+*^ mice and spine density (**f**) is significantly increased in both *Khsrp*^*−/−*^ and *Khsrp*^*+/−*^ compared to *Khsrp*^*+/+*^ mice. Density of stubby spines is significantly increased in both *Khsrp*^*−/−*^ and *Khsrp*^*+/−*^ while the density of mushroom spines is significantly increased in *Khsrp*^*−/−*^ compared to *Khsrp*^*+/+*^ mice (**g**). Error bars represent SEM (*N* = 5; *p < 0.05 by ANOVA) [Scale bars = **a** left panel, 500 and right three panels 200 µm; **b** 50 µm; **c** 10 µm; and **d** 2 µm].

Given the increase in mRNAs encoding proteins that can affect neuronal morphology and axon growth in the *Khsrp*^−/−^ mice observed above, we asked if loss of KHSRP expression changes neuronal morphology in vivo. For this, we crossed *Khsrp* knockout mice with Thy1-GFP mice that have GFP expression restricted to a subset of pyramidal neurons^24^. As a measure of axonal growth, we focused on the length of the hippocampal infrapyramidal mossy fiber bundle (IPB), a tract that is developmentally pruned after postnatal day 20 under normal conditions^25^. Therefore, adult 2-4-month-old mice were used for all morphological studies. IPB length is significantly increased in *Khsrp*^−/−^ compared to *Khsrp*^*+/+*^ in adult mice (Fig. 2a, e). We had previously shown that adult HuD overexpressing mice have an increase in IPB length^26^. Thus, loss of KHSRP has similar effects to the overexpression of the mRNA stabilizer HuD, suggesting that ratio of KHSRP to HuD is critical for regulating axonal growth.

To determine if loss of KHSRP affects dendrite development, we examined the apical dendrites of layer 5 pyramidal neurons in the somatosensory cortex from adult *Khsrp*^−/−^, *Khsrp*^+/−^ and *Khsrp*^*+/+*^ mice (Fig. 2b). The *Khsrp*^−/−^ and *Khsrp*^+/−^ mice showed a significant increase in apical dendrite spine density compared to *Khsrp*^+/+^ mice (Fig. 2c, d, f). With *Neurolucida* software, we defined the morphology of these dendritic spines as ‘mushroom’, ‘thin’, or ‘stubby’ shape that roughly correspond to their maturity^27^. Significantly greater numbers of stubby- and mushroom-shaped spines were observed in *Khsrp*^*−/−*^ mice compared to *Khsrp*^*+/+*^ (Fig. 2g). In addition, *Khsrp*^*+/−*^ mice showed significantly more stubby spines compared to *Khsrp*^*+/+*^ mice, with density of mushroom spines in *Khsrp*^*+/−*^ mice being intermediate between the *Khsrp*^*+/+*^ and *Khsrp*^*−/−*^ mice but not reaching statistical significance (Fig. 2g).

### Elevated neurite growth and KHSRP-target mRNAs with KHSRP deficiency are neuron-intrinsic

**Fig. 3:**
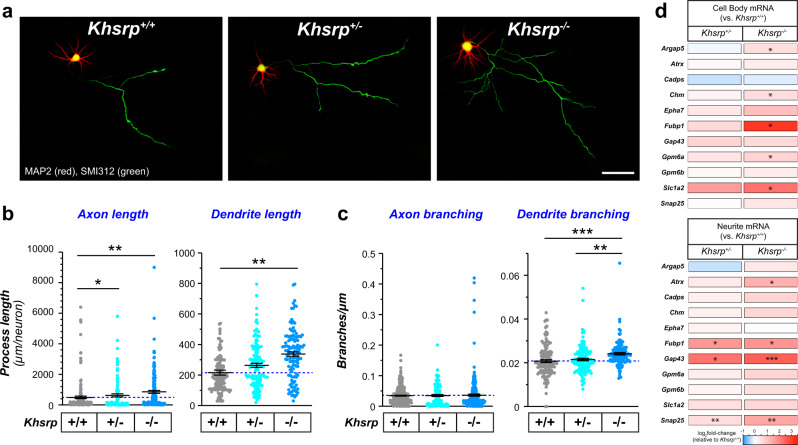
Neocortical neurons cultured from KHSRP deficient mice show increased axonal and dendritic growth. **a** Representative micrographs for neocortical neurons from *Khsrp*^*+/+*^, *Khsrp*^*+/−*^, and *Khsrp*^*−/−*^ littermates shown for DIV7 cultures as indicated. **b**, **c** Axon and dendrite lengths (**b**) and branch densities (**c**) are displayed as means ± SEM (*N* ≥ 75 neurons analyzed over at least three separate cultures preparations; *p ≤ 0.05, **p ≤ 0.001, and ***p ≤ 0.005 by one-way ANOVA with Tukey post-hoc). **d** Heat map for levels of KHSRP-target mRNAs linked to axon growth and neuronal morphology for cell body and neurite RNA analyses. Data are displayed as log_2_ fold-change relative to *Khsrp*^*+/+*^ cortical cultures (*N* ≥ 4 culture preparations; *p ≤ 0.05, **p ≤ 0.001, and ***p ≤ 0.005 vs. *Khsrp*^*+/*^ by two-tailed Student’s *t* test). See Supplementary Fig. 3 for parallel analyses in hippocampal neuron cultures and Supplementary Data 5 for mRNA copy number from these analyses [Scale bar = 100 µm].

Since KHSRP is also expressed in non-neuronal cells in the brain^9^, the in vivo neuron morphology and mRNA level changes in the *Khsrp*^*−/−*^ mice seen above could result from extrinsic effects on neurons. To explore this possibility, we used primary neuron cultures from single embryonic day 18 (E18) mouse embryos of *Khsrp*^*+/−*^ crosses that included *Khsrp*^−/−^, *Khsrp*^+/−^, and *Khsrp*^*+/+*^ genotypes in each litter. These cultures contain ≥95% neurons, allowing us to assess neuron-intrinsic growth mechanisms. Low density cultures analyzed at 7 days in vitro (DIV) were used to quantify axon and dendrite growth in the neurons by immunostaining using definitive markers for axons and dendrites. Axons were significantly longer in both neocortical and hippocampal neurons from *Khsrp*^−/−^ compared to *Khsrp*^*+/+*^ embryos (Fig. 3a, b; Supplementary Fig. 4a, b). Neocortical and hippocampal neurons from *Khsrp*^*+/−*^ mice showed axon lengths intermediate between *Khsrp*^−/−^ and *Khsrp*^*+/+*^, but this only reached significance for the *Khsrp*^*+/−*^ vs. *Khsrp*^*+/+*^ neocortical neurons (Fig. 3b; Supplementary Fig. 4b). The *Khsrp*^*−/−*^ hippocampal neurons showed more axon branching than those from *Khsrp*^*+/+*^ embryos (Supplementary Fig. 3c), but this was not seen in the neocortical neurons (Fig. 3c). *Khsrp*^−/−^ neocortical and hippocampal neurons showed significantly longer and more branched dendrites compared to *Khsrp*^*+/+*^ neurons (Fig. 3b, c; Supplementary Fig. 4b, c). Despite these changes in axon and dendrite growth with loss of KHSRP, neither the *Khsrp*^*−/−*^ or *Khsrp*^*+/−*^ neurons showed any significant difference in cell body diameters compared to the *Khsrp*^*+/+*^ neurons (average ± SEM: *Khsrp*^*+/+*^ = 203 ± 17, *Khsrp*^*+/−*^ = 197 ± 16, and *Khsrp*^*+/+*^ = 220 ± 19 µm).

With these changes in axonal and dendritic growth in cultures from mice with partial or complete loss of KHSRP, we asked if the mRNAs showing significant elevations in brain tissues of *Khsrp*^*−/−*^ mice from Fig. 1e might also be altered in neocortical neurons cultured from those mice. Further, since several of those KHSRP-target mRNAs are known to be transported into dendrites and/or axons, we separated cell bodies from neurites in these cultures to gain an assessment of overall and neurite-localized mRNA levels. *Argap5, Chm, Gpm6a Fubp1, Gap43*, and *Slc1a2* mRNAs were significantly increased in cell body RNA preparations from *Khsrp*^*−/−*^ neuron cultures; *Atrx, Fubp1*, *Gap43* and *Snap25* mRNAs were also elevated in *Khsrp*^−/−^ neurites (Fig. 3d; Supplementary Data 7). Interestingly, *Atrx* and *Snap25* mRNA showed no significant changes in the cell body RNA preparations, but the mRNAs were significantly elevated in the neurites of the *Khsrp*^*−/−*^ compared to *Khsrp*^*+/+*^ neuron cultures (Fig. 3d). *Gap43* and *Snap25* mRNAs have previously been reported to localize into axons of cultured neurons^19,28^. *Fubp1, Gap43*, and *Snap25* mRNAs were also increased in the *Khsrp*^*+/−*^ vs. *Khsrp*^*+/+*^ neurites, but none of the mRNAs tested show significant differences between *Khsrp*^*+/−*^ vs. *Khsrp*^*+/+*^ in the cell body RNA preparations (Fig. 3d; Supplementary Data 7). Overall, these data support that the increases in KHSRP-target mRNAs and axon dendrite growth seen in *Khsrp*^*−/−*^ mice arise, at least in part, from changes in neuronal gene expression via loss of the neuronal functions of KHSRP.

**Fig. 4:**
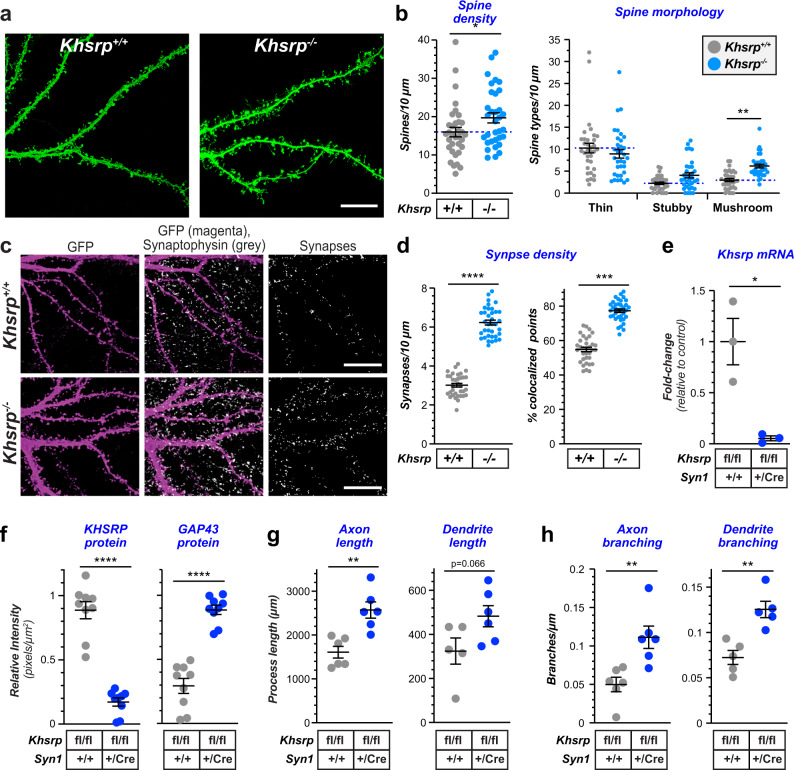
Altered neuronal growth is through neuron-intrinsic functions of KHSRP. **a** Representative epifluorescent images of distal dendrites for DIV23 cultures of LV-GFP transduced neocortical neurons from E18 *Khsrp*^*+/+*^ and *Khsrp*^*−/−*^ mice shown as indicated. **b** Quantification of dendritic spine density and morphology for cortical neurons as in a is shown (*N* > 30 neurons in at least 3 separate cultures; *p ≤ 0.05 and **p ≤ 0.01 by Student’s *t* test). **c** Representative epifluorescent images of distal dendrites for DIV23 cortical neuron cultures immunostained for GFP (magenta) and synaptophysin (gray). **d** Quantification of synapse density and % colocalization of pre- and post-synaptic markers for cortical neurons as in **c** is shown (*N* ≥ 30 neurons in at least 3 separate culture preparations; ***p ≤ 0.005 and ****p ≤ 0.001 by Student’s *t* test). **e**, **f** Quantification of *Khsrp* mRNA by RTddPCR and KHSRP and GAP43 proteins by immunofluorescence in E18 neocortical neurons cultured from *Khsrp*^*fl/fl*^ and *Khsrp*^*fl/fl*^ × *Syn1:Cre* mice is shown (*N* ≥ 30 neurons in at least 3 separate culture preparations; ****p ≤ 0.001 by Student’s *t* test). **g**, **h** Quantitation of axon and dendrite length (**g**) and branching (**h**) for neocortical neurons from E18 *Khsrp*^*fl/fl*^ and *Khsrp*^*fl/fl*^ × *Syn1:Cre* mice shown as indicated. Axons were identified by immunostaining for SMI312 and dendrites by Tau as in Fig. 3 (*N* ≥ 60 neurons over 3 separate culture preparations; **p ≤ 0.01 by Student’s *t* test).

We used DIV 23 cultures of E18 cortical and hippocampal neurons to measure potential neuron-intrinsic effects of KHSRP on dendritic spine formation. Dendrites and spines were visualized by expression of GFP to fill the neuronal cytoplasm for imaging. Both neocortical and hippocampal neurons from *Khsrp*^*−/−*^ mice showed a significant increase in dendritic spine density compared to *Khsrp*^*+/+*^ neurons (Fig. 4a, b; Supplementary Fig. 4d, e). An increase in mushroom-shaped spines in the *Khsrp*^*−/−*^ cortical neurons accounted for this difference in cortical neurons (Fig. 4b), while the *Khsrp*^*−/−*^ hippocampal neurons showed a significant increase in thin spines compared to *Khsrp*^*+/+*^ neurons (10.47 ± 0.79 vs. 8.28 ± 0.60 thin spines /10 µm; *p* ≤ 0.01). Consistent with increased spine density, both neocortical and hippocampal neurons from *Khsrp*^*−/−*^ mice showed increased synaptic density compared to those from *Khsrp*^*+/+*^ mice (Fig. 4c, d; Supplementary Fig. 4f).

To definitively test for neuron-intrinsic functions of KHSRP, we generated a mouse with loxP sites between exons 1 and 2 and exons 6 and 7 of the *Khsrp* gene (*Khsrp*^*fl/fl*^). These mice were crossed with the neuronal specific Syn1:Cre driver line to generate a neuronal specific knockout of *Khsrp* (*Khsrp*^*fl/fl*^ × *Syn1:Cre*). *Khsrp* mRNA was clearly depleted from embryonic neocortical neurons cultured from the *Khsrp*^*fl/fl*^ × *Syn1:Cre* mice (Fig. 4e). By immunofluorescence, KHSRP protein signals were markedly decreased and GAP43 protein signals were increased in these neuron-specific KHSRP knockout cultures (Fig. 4f). Neocortical neurons cultured from these *Khsrp*^*fl/fl*^ × *Syn1:Cre* mice also showed increased axon and dendrite lengths and branching consistent with the alterations seen in neurons from the *Khsrp*^*−/−*^ mice, but notably dendrite lengths did not reach statistical significance (Fig. 4g, h). Consistent with increased synapse density in the cultures of KSHRP-depleted neurons, somatosensory cortex of both *Khsrp*^*−/−*^ and *Khsrp*^*fl/fl*^ × *Syn1:Cre* mice show increased synapse density compared to *Khsrp*^*+/+*^ mice (Supplementary Fig. 5). Furthermore, the cortex from *Khsrp*^*+/fl*^ × *Syn1:Cre* mice shows synapse numbers intermediate between wild type and KHSRP knockout samples (Supplementary Fig. 5b). Taken together, these data point to a neuron-specific phenotype altering axon and dendrite growth and synaptogenesis that occurs upon loss of KHSRP.

### Loss of KHSRP elevates excitatory neurotransmission

**Fig. 5:**
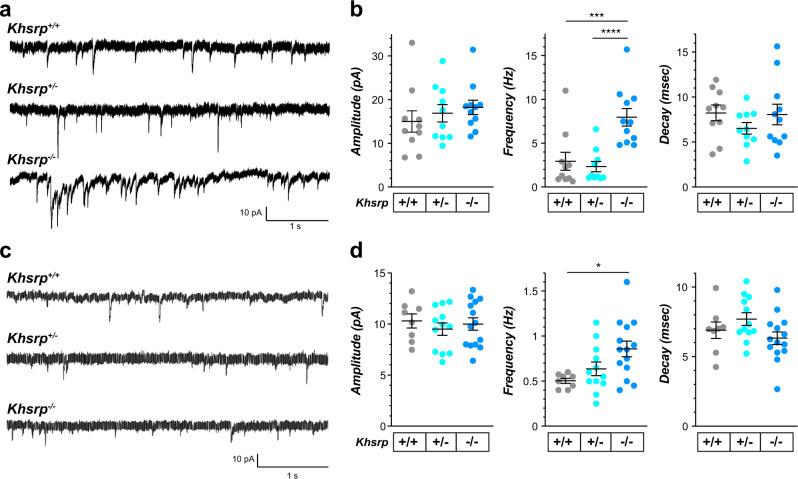
Increased pre-synaptic activity in KHSRP deficient mice. **a** Representative traces of AMPA receptor-mediated miniature excitatory post-synaptic currents (mEPSCs) in CA3 pyramidal neurons of the dorsal hippocampus. **b** mEPSC frequency is significantly increased in *Khsrp*^−/−^ relative to *Khsrp*^+/+^ and *Khsrp*^+/−^ hippocampus. There were no significant differences in mEPSC amplitude or duration (decay time) between genotypes (*N* = 10 *Khsrp*^+/+^, 10 *Khsrp*^*+/−*^, and 11 *Khsrp*^*−/−*^ over 5 animals per genotype; frequency data analyzed by one-way ANOVA, F(2,28)=11.97, *p* = 0.0002; ***p ≤ 0.01 and ****p ≤ 0.001 by Tukey’s post-hoc comparisons). **c** Representative traces of AMPA receptor-mediated mEPSCs in layer 5 pyramidal neurons of the infralimbic cortex. **d** Infralimbic cortical neurons show increased mEPSC frequency in the *Khsrp*^−/−^ relative to *Khsrp*^+/+^ mice. As in the CA3, no differences in mEPSC amplitude or duration were detected (*N* = 8 *Khsrp*^+/+^, 12 *Khsrp*^*+/−*^, and 14 *Khsrp*^*−/−*^ over 5 animals per genotype; frequency data analyzed by one-way ANOVA, *F*(2,31)=5.03, *p* = 0.013; *p ≤ 0.05 by Tukey’s post-hoc comparisons).

The changes in neuronal morphology and increased levels of KHSRP-target mRNAs encoding synaptic proteins in the KHSRP knockout neurons seen above raise the possibility that loss of KHSRP could alter synaptic function. We used brain slice electrophysiology to compare synaptic function between *Khsrp* knockout and wild type mice. For this, we initially measured AMPA/kainate receptor-mediated mEPSCs in CA3 pyramidal neurons of the dorsal hippocampus of adult *Khsrp*^*−/−*^, *Khsrp*^*+/−*^ and *Khsrp*^*+/+*^ mice. mEPSC frequency was significantly increased in CA3 hippocampal neurons from *Khsrp*^*−/−*^ compared to *Khsrp*^*+/+*^ and Khsrp^+/−^ mice (Fig. 5a, b). Since the analyses of neurite growth and KHSRP-target mRNAs showed some differences between hippocampal and cortical neurons, we also assessed synaptic function in infralimbic cortex layer V neurons. The *Khsrp*^*−/−*^ cortex again showed a significant elevation of mEPSC frequency compared to those of *Khsrp*^*+/+*^ brains (Fig. 5c, d). The *Khsrp*^*+/−*^ cortical neurons showed average frequency intermediate between *Khsrp*^*−/−*^ and *Khsrp*^*+/+*^ cortices, but this did not reach statistical significance. There was no difference in mEPSC amplitudes and mEPSC durations between the genotypes in either hippocampal CA3 or infralimbic cortex (Fig. 5b, d). Changes in mEPSC frequency traditionally indicate involvement of pre-synaptic, rather than post-synaptic, mechanisms^29^. Thus, the increased mEPSC frequency in the *Khsrp*^*−/−*^ mice likely reflects increased numbers of functional synapses, consistent with observed increases of axon and dendrite growth and synapse density in those mice. Interestingly, post-synaptic sensitivity to glutamate release (e.g., number of synaptic AMPA receptors, AMPA receptor subunit composition), as reported by mEPSC amplitude and duration, is not altered by the loss of KHSRP.

### Loss of KHSRP increases locomotor activity and impairs hippocampal- and prefrontal cortex-dependent memory consolidation

Given the alterations in neuronal morphology and function, we asked whether loss of KHSRP expression would affect mouse behavior. Initial behavioral screening was adapted from a subset of tests derived from the Irwin screen for physical health, appearance, sensory utility, motor coordination, and neurological function^30–32^. As shown in Supplementary Data 8, *Khsrp*^+/−^ and *Khsrp*^−/−^ mice exhibit normal physical features including weight, whiskers, eyes, eyelids, teeth, tail, and fur. They also show no differences in normal mouse behaviors including gait, grooming, and rearing. However, *Khsrp*^−/−^ mice display significantly increased circling and spontaneous running (Supplementary Data 8), which may indicate a level of hyperactivity from deletion of the *Khsrp* gene.

**Fig. 6:**
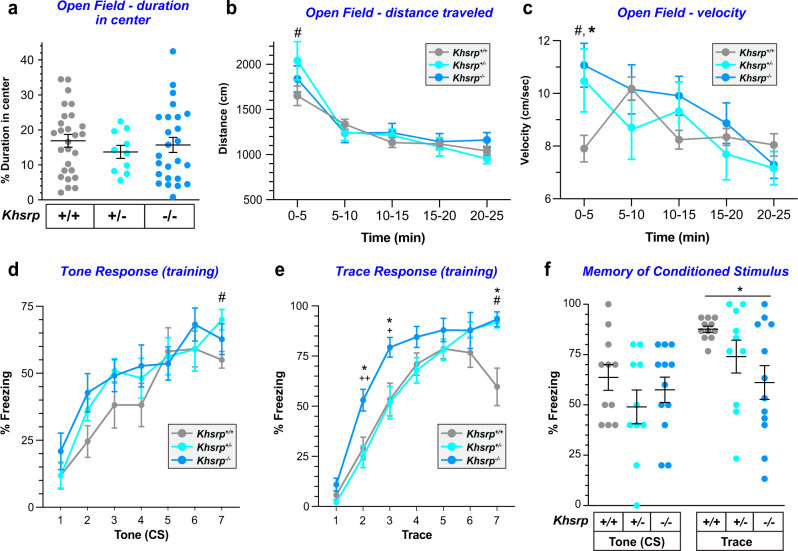
KHSRP deficient mice show increased locomotor activity and impaired hippocampal-dependent memory consolidation. **a** Mice with decreased KHSRP levels display no difference in percentage of time in the center in the Open field test. **b**, **c** *Khsrp*^*−/−*^ and *Khsrp*^*+/−*^ mice have increased locomotor activity in the first 5 min for day 1 as measured by distance traveled (**b**) or velocity (**c**) but decreased locomotor activity over time all values are mean ± SEM (*N* = 28 for *Khsrp*^*+/+*^ and *Khsrp*^−/−^ and n = 10 for *Khsrp*^*+/−*^; repeated measures ANOVA show a significant time × genotype interaction *p* = 0.0026). Tukey post-hoc tests revealed that the first bin (0–5 min) is significantly different from all other time points for both distance traveled and velocity. This difference is driven by the *Khsrp*^*+/−*^ mice for distance traveled (ANOVA *F*(2,60) = 0.2306, *p* = 0.0449, ^#^*p* ≤ 0.05 *Khsrp*^*+/−*^ vs. *Khsrp*^*+/+*^), while for velocity *Khsrp*^*+/−*^ and *Khsrp*^−/−^ animals are both significantly elevated compared to *Khsrp*^*+/+*^ (ANOVA *F*(2, 60)=1.895, *p* = 0.0064, ^##^*p* ≤ 0.01 *Khsrp*^*+/−*^ vs. *Khsrp*^+/+^ and *p* = 0.0442, *p ≤ 0.05 *Khsrp*^−/−^ vs. *Khsrp*^*+/+*^). **d**, **e** All mice demonstrate increased freezing during training with increasing presentations of the conditioned stimulus (CS; tone, **d**) and the trace interval following CS (**e**). Notably, *Khsrp*^*+/−*^ mice show increased freezing vs. *Khsrp*^*+/+*^ mice at the last tone and trace interval presentation. *Khsrp*^−/−^ mice also show increased freezing during the trace period during training vs. *Khsrp*^*+/+*^ and *Khsrp*^*+/−*^. Data are shown as mean ± SEM, *n* = 11 (6 male and 5 females/genotype; ^+^*p* ≤ 0.05 and ^++^*p* ≤ 0.01 for *Khsrp*^*+/−*^ vs. *Khsrp*^*−/−*^mice, and *p ≤ 0.05 *Khsrp*^−/−^ vs. *Khsrp*^*+/+*^ and ^#^*p* ≤ 0.05 *Khsrp*^*+/−*^vs. *Khsrp*^*+/+*^ by repeated measures ANOVA followed by one-way ANOVA for each time point). **f** All mice freeze equally during CS presentation approximately 24 h after training, but *Khsrp*^−/−^ mice display decreased freezing during the trace interval compared to *Khsrp*^*+/+*^. Data are shown as mean ± SEM, *n* = 11 (6 male and 5 females/genotype; one-way-ANOVA (*F*(2,27) = 3.948, *p* = 0.0149, *p ≤ 0.05). See Supplementary Fig. 6 for sex-specific responses of each genotype.

We next assessed anxiety-like behavior, locomotion, and exploratory tendencies in these mice using the novel open field and elevated zero maze tests. *Khsrp*^−/−^ and *Khsrp*^+/−^ mice showed no significant differences in percent of time in the center of the open field (Fig. 6a) or in the open arm duration on the zero maze compared to *Khsrp*^*+/+*^ mice (Supplementary Fig. 6a), which together indicate no alteration in anxiety-like behaviors with KHSRP loss. However, we found a significant effect of time in both distance and velocity in the open field test (Fig. 6b, c), which were due to increased velocity and distance traveled for the *Khsrp*^−/−^ and *Khsrp*^+/−^ vs. *Khsrp*^*+/+*^ mice on day 1 compared to all other days. Further examination of day 1 revealed that the first-time bin (0–5 min) was significantly different from all other time points for both distance traveled and velocity, which is indicative of novelty-induced locomotion. This difference is driven by the *Khsrp*^+/−^ mice for distance traveled, while the velocity of *Khsrp*^−/−^ and *Khsrp*^+/−^ mice was significantly elevated compared to *Khsrp*^*+/+*^ controls (Supplementary Fig. 6b, c).

Considering the elevations in hippocampal axon/dendrite growth, KHSRP-target mRNAs, and synaptic activity with loss of KHSRP, we next used trace conditioning to test hippocampal function in the *Khsrp*^−/−^ and *Khsrp*^+/−^ mice^33^. Trace conditioning tests the ability to associate a conditioned stimulus (CS) to an unconditioned stimulus (US) separated by a 30 s trace interval. Training consisted of seven tone (CS) and shock (US) presentations, with initial learning of the task assessed by percentage of freezing during the CS and trace interval. All mice progressively increased freezing to the CS and during the trace interval following each CS presentation during training. While both *Khsrp*^+/−^ and *Khsrp*^−/−^ took 7 training sessions to achieve maximal freezing behavior, as expected^34^, the *Khsrp*^*+/+*^ mice reached maximal freezing after fewer sessions (Fig. 6d, e). There were no main effects of genotype, sex, nor a sex × genotype interaction during the CS and trace portions of training (Supplementary Fig. 6d), so the male and female data for individual genotypes were combined for subsequent analyses. *Khsrp*^−/−^ mice showed significantly higher freezing than the *Khsrp*^*+/+*^ mice at the second, third, and seventh trace periods and significantly higher freezing than the *Khsrp*^+/−^ at the second and third periods. (Fig. 6e). To assess retention of trace fear conditioned responses, freezing behavior of *Khsrp*^+/−^ and *Khsrp*^−/−^ mice was evaluated 24 h post training. The CS was delivered in a novel context to examine the response independent of the original fear context. We found no significant effects of genotype on percent freezing to the CS; however, *Khsrp*^−/−^ mice displayed decreased freezing during the trace interval compared to *Khsrp*^*+/+*^ mice, which was observed in both male and female mice (Fig. 6f; Supplementary Fig. 6e). Taken together, these changes in trace conditioning suggest that deletion of KHSRP impairs memory consolidation.

We next used novel object recognition (NOR)^35,36^, a different hippocampal-dependent memory test that also requires intact cortical function^37–39^, to further evaluate learning and memory in mice of the three different genotypes. Two-way ANOVA of genotype × sex interactions in the % recognition index (RI) showed a significant effect of genotype but no effect of sex; therefore, animals of both sexes were combined for the analyses. The *Khsrp*^−/−^ mice showed a significant increase in RI compared to *Khsrp*^*+/+*^ mice without any preferences in object location (Supplementary Fig. 7a, b). Although this finding was initially unexpected, further analyses of the behavior of *Khsrp*^−/−^ mice revealed they also displayed increased duration spent at and frequency of visits to the novel object, as well as increased total distance traveled and velocity during the test phase compared to *Khsrp*^*+/+*^ mice (Supplementary Fig. 7c–e). Although the NOR has been traditionally used to detect hippocampal deficits, this test also requires intact function of several cortical regions including the prefrontal cortex^37,39,40^.

**Fig. 7:**
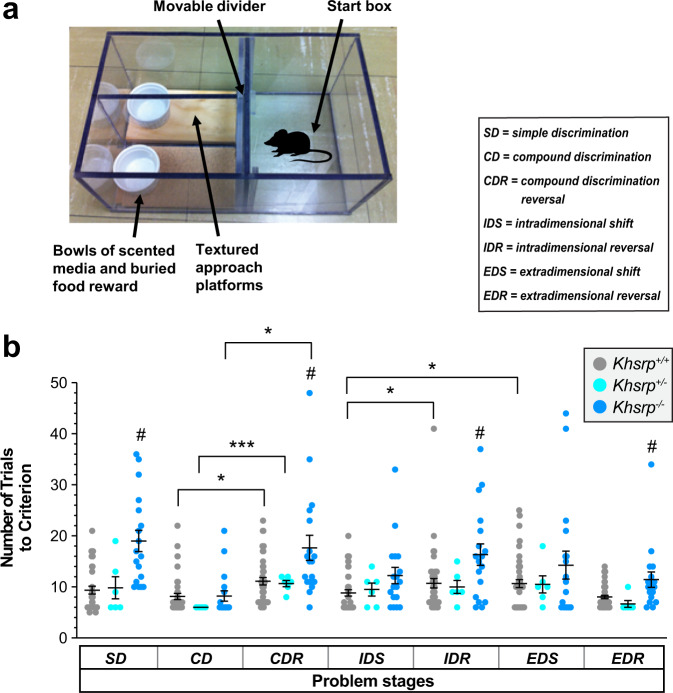
KHSRP deficient mice show decreased prefrontal cortical function in the ASST test. **a** Diagram of the chamber used for all ASST trials and list of specific problem stages (see Supplementary Fig. 8a for problem stages and stimuli). **b** All mice were able to perform discrimination (SD, CD, and IDS) and reversals (CDR, IDR, EDR), and showed increased number of trial for CDR vs. CD, although the reversals were no more difficult for *Khsrp*^*+/−*^ and *Khsrp*^*+/−*^ mice than the preceding stage for IDR vs IDS. Only *Khsrp*^*+/+*^ mice formed an attentional set (EDS vs. IDS) while *Khsrp*^*+/−*^ and *Khsrp*^*+/−*^ did not (*N* ≥ 10/genotype. Data were analyzed by Brown-Forsythe One-way ANOVA tests and Welch’s *t* tests; *p ≤ 0.05, **p ≤ 0.01, and ***p ≤ 0.001; note error is reported for all values and the column without bars has an error of 0).

Considering the NOR results and our finding that *Khsrp*^−/−^ mice showed increased synaptic excitability in infralimbic cortex, we next used the attentional set shifting task (ASST) to assess the effect of loss of KHSRP in frontal cortex function. It is well established that attentional set shifting is mediated by regions of the ventromedial prefrontal cortex (vmPFC), which includes the infrapyramidal cortex^41–43^ while reversal learning is mediated by the orbitofrontal cortex (OFC)^44–46^. The ASST tests executive function by initially exposing animals to a series of problem stages that are used to predict food location^41^. This includes discrimination-reversal learning within one dimension (odor or tactile discrimination within a platform), an intra-dimensional shift (IDS) with novel exemplars within the previously learned dimension (i.e., odor 1 to odor 2 or platform 1 to platform 2), as well as an extra-dimensional shift (EDS) to the previously unrewarded dimension (i.e., odor to platform). Figure 7a depicts the ASST chamber where mice number is based upon specific odor or platform cues presented at each stage as a predicter for food location (Supplementary Fig. 8). The cues used for all the ASST stages with odor as starting dimension are shown in Supplementary Fig. 8a. It is well-known that tactile learning is more difficult to initially acquire than olfactory learning^47^. Consistent with this, we found a significant main effect of starting dimension during the Simple Discrimination (SD) and Compound Discrimination (CD) stages that was eliminated by the Compound Discrimination Reversal (CDR) stage (Supplementary Fig. 8b). Since mice of the three genotypes were able to learn to efficiently discriminate tactile differences after repeated exposures, the ASST test was used for stages of increasing difficulty. As shown in Fig. 7b, *Khsrp*^*+/+*^ mice formed an attentional set as measured by increased numbers of trials to criteria in the EDS vs. IDS stages, but neither *Khsrp*^+/−^ nor *Khsrp*^−/−^ mice established an attentional set, indicating a frontal cortical dysfunction in these mice. In addition, compared to wild-type mice, both *Khsrp*^+/−^ and *Khsrp*^−/−^ mice required increased number of trials in the first reversal (CDR vs. CD) but this was not sustained on the second reversal (IDR vs. IDS). Furthermore, *Khsrp*^−/−^ mice show significantly increased number of trials to criteria for the SD, CDR, IDR and EDR stages. Taken together, these observations indicate that *Khsrp*^−/−^ mice are able to perform each discrimination, but they do not form an attention shift set so that they approach any changes in food prediction cues as completely new cues, independently of their previous choices.

## Discussion

It is increasingly clear that post-transcriptional regulation of mRNAs plays a dynamic role in the regulation of neuronal genes and subsequent changes in behavior^2,48^. Critically, RBPs can regulate the stability of bound mRNAs^2,49^. Since one mRNA can be translated into protein many times over, regulating the stability of mRNAs can dynamically modify cellular protein content. Here, we show that loss of the RBP KHSRP leads to a unique set of electrophysiological and behavioral changes, resulting in decreased hippocampal- and frontal-dependent learning and memory. The ‘KHSRP-target mRNAs’ reported here were identified by integrating mRNAs showing enrichment in KHSRP pull downs from wild type mouse brain (by RIP-Seq) and increased levels in brains of KHSRP deficient mice (by microarray). Since we used mRNAs rising to significant cut-offs across two different detection methods, our approach cannot distinguish changes in different mRNA isoforms nor differences in affinities for binding to KHSRP. Future studies will need to address these variables. Despite these limitations, the increased levels of KHSRP-target mRNAs in brain tissues and parallel changes in KHSRP-target mRNAs plus alterations in axon and dendrite growth in vivo and in cultured neurons, the changes in neural activity and behavior seen in the *Khsrp*^*−/−*^ mice are undoubtedly driven by neuron-intrinsic elevations in KHSRP-target mRNAs when KHSRP expression is decreased. Analyses of neuronal-specific knockout of *Khsrp* confirms a cell-intrinsic disruption of neuronal growth and gene expression upon loss of neuronal KHSRP expression.

We previously reported that loss of KHSRP leads to aberrant axonal outgrowth in DIV5 cultured embryonic cortical neurons due to increase in the levels of *Gap43* mRNA^12^, a mRNA that is post-transcriptionally regulated by changes in its stability^50,51^. Correct axonal growth regulation is critical for proper development and maintenance of neuronal networks, and the work here shows that the axon growth abnormalities are maintained into more mature neurons, with clearly defined axonal and dendritic polarity, as well as impact dendrite growth and spine formation in vitro and in vivo. Since overexpression of GAP-43 also leads to excessive axonal growth^26,52^, it is intriguing to speculate that mRNA destabilizing RBPs like KHSRP are needed to control levels of neuronal growth-associated mRNAs as neurons need to slow their growth when they start to make synaptic connections. Interestingly, we found that 444 of the neuronal KHSRP targets identified by RIP-seq were also up-regulated in the cortex of *Khsrp*^−/−^ mice and contain 3′ UTR AREs for KHSRP binding (Supplementary Data 3). Thus, binding by KHSRP is predicted to shorten half-lives of those target mRNAs. Many of the KHSRP-target mRNAs identified herein encode proteins involved in neuronal development, axon growth, and synaptic plasticity. Our analyses confirm that absence of KHSRP, with stabilization of these target mRNAs, specifically increases axonal and dendritic growth as well as increases density of dendritic spines. The subtypes of spines that are increased in KHSRP deficient brains include both mature (mushroom) and more immature or plastic (stubby) spines, with density of stubby spines significantly increased in both *Khsrp*^*+/−*^ and *Khsrp*^*−/−*^ mice. Changes in axonal and dendrite length and branch points were also present in primary neuronal cultures from the *Khsrp*^*−/−*^ *mice*, indicating that KHSRP’s regulation of neuronal mRNAs is responsible for these changes.

The increased mEPSC frequency without changes in amplitude or decay seen in the hippocampus and prefrontal cortex of the *Khsrp*^*−/−*^ mice is consistent with increased numbers of synaptic terminals, increased pre-synaptic neurotransmitter release, or both^29^. The KHSRP-target mRNA *Gap43*, which encodes a well-known growth-promoting protein that localizes to axonal growth cones^53^, has previously been implicated in hippocampal-dependent learning^54^. In contrast to our findings, in vivo overexpression of GAP43 enhanced learning^55^. This suggests that overall increased GAP43 expression seen with loss of KHSRP cannot alone explain the changes in electrophysiological or behavioral properties of *Khsrp*^*−/−*^ mice. This is not unexpected, as the overall phenotype of the KHSRP knockout mice is undoubtedly driven by the sum of the neuronal proteome changes resulting from elevations in KHSRP-target mRNAs in these mice. Previous studies suggested that axonal *Gap43* mRNA translation contributes to elongating axonal growth^56^, and loss of KHSRP increases both axon length and branching in the present study. Notably, the transgenic mice used by Routtenberg et al.^55^ for GAP43 overexpression only included a few nucleotides of the 3′UTR^52^, and did not include the ARE that we have previously shown is needed for *Gap43* mRNA’s axonal localization^57^. Consequently, increase in locally synthesized GAP-43 could bring a different behavioral phenotype than seen by Routtenberg et al.^55^. Consistent with this, we previously only observed increased axonal growth when the overexpressed *Gap43* mRNA was targeted into axons through its 3′UTR^56^.

Other KHSRP-target mRNAs encode proteins linked to axon growth and synaptogenesis, including ARGAP5, ATRX, CADPS, CHM, EPHA7, FUBP1, FUBP3, GPM6A, GPM6B, RAC1, SLC1A2, and SNAP25 (see Supplementary Data 4), that could affect numbers of synaptic terminals as evidenced by increased dendritic spine and synapse density in the brains and cultured neurons of the *Khsrp*^*−/−*^ mice. Increased neurotransmitter release could also be driven by KHSRP-target mRNA encoded proteins, as mRNA elevations for the SNARE protein SNAP25 seen with loss of KHSRP could elevate the probability of synaptic vesicle release^58^. Interestingly work from the Hengst lab has linked axonally localizing *Snap25* mRNA to formation of pre-synaptic terminals^19^, and we see increased levels of *Snap25* mRNA in neurites of the *Khsrp*^*−/−*^ and *Khsrp*^*+/−*^ mice. These observations raise the possibility that localized modulation of mRNA survival by KHSRP in axons helps to sculpt synaptic connectivity and contribute to synaptic plasticity in the brain. Consistent with this notion, recent work from the Schuman lab has shown an unexpectedly large population of proteins translated in pre-synaptic terminals of the adult brain including both *Gap43* and *Snap25* mRNAs^59^. It is intriguing that *Fubp1* and *Fubp3* mRNAs are identified as KHSRP targets, as these RBPs are in the same protein family as KHSRP^6^. Previously published affinity proteomics for RBPs in PNS axons identified KHSRP and FUBP1 as binding to *Gap43* mRNA’s ARE^60^. FUBP1 has been shown to regulate mRNA stability and play a role in mRNA translation^61,62^, including IRES-dependent translation that antagonizes KHSRP in non-neuronal cells^63^. Thus, it is intriguing to speculate that upregulation of other FUBPs may contribute to the neural phenotype seen with loss of KHSRP.

Similar to findings here in KHSRP knockout mice, we previously showed that mice overexpressing the RNA-binding protein HuD have elevated levels of ARE-containing mRNAs as well as altered neuronal morphology and associative learning^26,64^. The trace fear conditioning used here requires an intact hippocampus to form an association between the CS and US^33^. The *Khsrp*^−/−^ mice are able to initially learn the association between the CS and US during training. However, they display decreased freezing to the CS approximately 24 h later, indicating that *Khsrp*^−/−^ mice have deficits in temporal processing of information due to the time separation between the training and testing^65,66^. This may indicate a deficit in hippocampal-dependent memory consolidation. In addition, mice with a deletion of KHSRP also show deficits in the ASST task, which requires functions of the ventromedial PFC. While *Khsrp*^−/−^ mice can perform reversals, these problems are no more difficult than the preceding discrimination indicating that these mice approach each problem as if it were novel. Thus, global loss of KHSRP impairs attentional set formation of species-appropriate stimuli.

Overall, our results indicate that KHSRP modulates levels of its target mRNAs required for the development of neural connectivity and potentially synaptic plasticity. Loss of KHSRP leads to significant changes in neuronal morphology through neuron-intrinsic mechanisms that persist into adulthood, resulting in impaired glutamatergic transmission and behaviors linked to functions of the hippocampus and prefrontal cortex. Our study emphasizes the importance of post-transcriptional regulation by KHSRP as a driver for brain development and function. The KHSRP-target mRNAs identified here show upregulation upon loss of KHSRP indicating they are targets for destabilization by KHSRP. However, it should be noted that KHSRP has functions beyond promoting mRNA decay, so the morphological, electrophysiological, and behavioral changes presented here for KHSRP deficient mice could be impacted by other roles of KHSRP. These observations point to KHSRP is a post-transcriptional master regulator of a mRNA regulon linked to brain development and function.

## Materials and methods

### Animals

All animal studies were conducted in accordance with guidelines for animal use and care established by the University of New Mexico Health Science Center and University of South Carolina Institutional Animal Care and Use Committees (IACUCs). The *Khsrp*^*−/−*^ animals have deletion of exons 1–13 as described in Lin et al.^67^ and were cross-bred with C57Bl/6 for at least 10 generations. For morphological studies, *Khsrp*^*−/−*^ mice and were cross-bred with *B6.Cg-Tg(Thy1-EGFP)OJrs/GfngJ* mice (termed Thy1-GFP herein; obtained from Jackson Labs) to eventually generate *Khsrp*^*−/−*^*, Khsrp*^*+/−*^ and *Khsrp*^*+/+*^ with GFP expression in select neurons.

C57Bl/6 mice with loxP sites in the *Khsrp* allele were generated by Biocytogen (Wakefield, MA) using CRISPR/EGE™-based gene editing. Based on sequence analyses of the mutant allele, a frameshift in *Khsrp* mRNA coding sequence would occur upon Cre-driven recombination. These mice were bred to homozygosity after crossing with wild type C57Bl/6 mice. *Khsrp*^*fl/fl*^ mice were bred to Syn1:Cre driver line (Jackson Labs, RRID:IMSR_JAX:003966) to generate neuronal specific knockout of *Khsrp*; only female Syn1:Cre mice and heterozygous floxed mice were used for mating to avoid germline transmission of the deleted allele. Expression of *Khsrp* mRNA and KHSRP protein were tested by RTddPCR, immunoblotting, and immunofluorescence.

Genotyping for constitutive *Khsrp* knockout was performed using PCR (see below) with primers spanning the exon 1 to exon 13 deletion of the mouse Khsrp gene^67^ or wild type sequence (Supplementary Data 9). For the conditional knockout, primers for the 5′ and 3′ loxP sites were used (forward and reverse primer pairs for each LoxP site; Supplementary Data 9). *Syn1:Cre* allele genotyping was performed using PCR with primers to recognize the Cre transgene as well as an internal positive control.

### Primary neuron cultures

Primary cortical neuron cultures were prepared from embryonic day 18 (E18) mice. Cortices and hippocampi were dissected in Hibernate E (BrainBits, IL) and dissociated using the *Neural Tissue Dissociation kit* according to manufacturer’s protocol (Miltenyi Biotec, Bergisch Gladbach, Germany). For this, minced neocortices or hippocampi were incubated in a pre-warmed Enzyme Mix 1 at 37 °C for 15 min; tissues were then triturated with blunted glass pipette and again incubated with Enzyme Mix 2 for 10 min. Triturated tissue was applied to a 40 µm cell strainer. After washing and centrifugation, neurons were seeded on polyethylene-tetrathalate (PET) membrane (1 µm pores; Corning, NY) inserts, glass coverslips or glass-bottomed multiwell plates. All culture substrates were pre-coated with poly-d-lysine (Sigma, MO). *NbActive-1 medium* (BrainBits) supplemented with 100 U/ml of Penicillin-Streptomycin (Life Technologies, MA), 2 mM L-glutamine (Life Technologies), and 1 X N21 supplement (R&D Systems, MN) was used as culture medium. Inserts were seeded at a density of 1.5 × 10^6^ cells per insert and glass-plated cultures were seeded at 15,000 cells per 12 mm coverslip or well of a 24 well plate.

Neurons cultured on glass were used for morphological analyses with durations in culture indicated in the results. Neurons cultured in the PET inserts were used for isolation of neurites from lower membrane surface with cellular material along upper membrane referred to as a cell body preparation as previously described^68^.

For visualizing dendritic spines in neuron cultures, cortical and hippocampal neurons were transduced with AAV8-GFP (UNC Viral Vector Facility, NC) at 18 days in vitro (DIV). Cultures were then fixed as above at DIV23 and analyzed by fluorescent microscopy for GFP.

### RNA isolation and analyses

Total RNA was extracted from the neocortex and hippocampus of male 2-4 month-old *Khsrp*^−/−^, *Khsrp*^*+/−*^ and *Khsrp*^*+/−*^ littermate mice using *Trizol* reagent (Invitrogen, CA). For analyses of RNAs from cultured neurons, cell body vs. neurite RNA was isolated using *RNAeasy Microisolation Kit* (Qiagen, CA).

Mouse genotyping was done by PCR using DNA extracted from ear punches taken at weaning (for maintenance of mouse lines and for experiments with adult mice) or from tissues harvested at time of culture preparations (for embryos). Aliquot of each DNA isolate was used for standard PCR reactions. Primers used for genotyping are shown in Supplementary Data 9. PCR products were analyzed by agarose gel electrophoresis with GelGreen Nucleic Acid Stain (Biotium, Fremont, CA).

For microarray analyses of transcript levels in cortices from adult males *Khsrp*^+/+^ vs. *Khsrp*^*−/−*^, mRNAs were purified after removal of rRNA (*mRNA-ONLY™ Eukaryotic mRNA Isolation Kit*, Epicentre Biotech., WI). Fluorescently labeled cRNAs derived from these transcripts were used to probe Agilent *Mouse V4.0 LncRNA Array* containing probes for 22,692 mRNAs (ArrayStar, Inc., MD).

Reverse-transcriptase (RT) coupled PCR was used to validate the KHSRP-target mRNAs identified from microarrays and RIP-Seq analyses (see below). All RTddPCR analyses were run on RNA isolates from at least 3 mice or culture preparations. For this, RNA yields were normalized across samples prior to reverse transcription based on fluorometric quantification using Ribogreen reagent (Invitrogen). 10–50 ng of RNA from brain samples or 10–25 ng of RNA from cell body and neurite preparations of neuron cultures was reverse transcribed using Sensifast (Bioline, TN). For the neuron cultures, single mouse pup cultures were performed from littermate mice with genotypes tested as above while the neurons were in culture (and analyses performed blinded to genotype). Tissues taken from mouse pups at the time of dissection was used for genotyping as outlined above. cDNA samples were diluted and then processed for extended cycle PCR (to test for neurite purity) or quantitative ddPCR. Extended cycle PCR, with primers for cell body (*cJun*) and glial contamination (*Gfap*) and *Map2* and *Actb* primers as positive control (i.e., neurite localizing mRNAs), was used to assess the purity of neurite RNA preparations. These PCR products were analyzed by agarose gel electrophoresis with GelGreen Nucleic Acid Stain. For ddPCR, we used Evagreen reagent (Bio-Rad, CA) with an automated droplet generator; after standard PCR cycles, droplets were analyzed using a QX200TM (Bio-Rad). Signals were normalized between reactions/samples using the mitochondrially encoded 12S ribosomal RNA (12S rRNA). Primer sequences are shown in Supplementary Data 9.

### KHSRP-target identification using RNA-immunoprecipitation-sequencing (RIP-seq)

RNA-immunoprecipitation (RIP) assays were performed in triplicates using anti-KHSRP antibodies (5 µg/mg lysate; Novus, CO, #NBP1-18910) pre-loaded onto protein G magnetic beads (ThermoFisher Sci., Walthan, MA) as described^69^. Eight sets of E18 cortices of mixes sexes from *Khsrp*^*+/+*^ mice were used for these assays and equal number of E18 cortices of *Khsrp*^*−/−*^ mice were used as controls for the RIP. After washing the beads, RNA was extracted using Trizol and sent for sequencing at the National Center for Genome Resources (NCGR, Santa Fe, NM). Sequencing libraries were generated using the TruSeq™ RNA Sample Preparation Kit (Illumina, San Diego, CA). The resulting cDNA was used for cluster generation and sequencing by synthesis on the Illumina HiSeq. Library preparation, sequencing, and statistical analysis was performed at NCGR. Sequencing reads were first filtered by removing adaptor sequences. The remaining reads were aligned and mapped to the mouse genome (M. musculus release 81). Reads were normalized for fragment and library size, and DESeq was used for differential expression analysis and multiple testing correction using a false-discovery rate cut-off of *q* < 0.05. Significantly enriched RNAs in *Khsrp*^*+/+*^ RIP-seq were identified using a log_2_-fold change (FC) enrichment of 1.4 (equivalent to a linear FC = 2.64).

### Bioinformatics analyses

The following filters were used to identify neuronal KHSRP targets that were up-regulated in *Khsrp*^*−/−*^ mice: (1) differentially expressed genes with adjusted *p*-values < 0.05 and fold change >1.25 in *Khsrp*^*−/−*^; and (2) RIP-seq data including targets significantly enriched in the KHSRP pull downs for *Khsrp*^+/+^ E18 cortices as described above. We next used this set, to search for the presence of destabilizing ARE motifs in their 3’ UTRs as described in Bolognani et al.^69^. Briefly, the presence of types I, II, typical AREs and type III, U-rich atypical AREs were identified using our own BioPerl scripts with consensus sequences for the three types of AREs^69^. Furthermore, IPA (Qiagen) was used to identify biological pathways and networks enriched in genes within the Nervous system development and function category. The data sets generated during the current study are available in the NCBI GEO repository (accession number = GSE166010).

### Immunofluorescent staining

All immunofluorescence steps were conducted at room temperature unless specified otherwise. Neuron cultures were fixed with 4 % paraformaldehyde (PFA) in phosphate-buffered saline (PBS) for 15 min and washed 3 times in PBS. Samples were permeabilized with 0.3% Triton X-100 in PBS for 15 min and blocked for 1 h in 5% BSA in PBS + 0.1% Triton X-100 (PBST). Samples were then incubated overnight in humidified chambers at 4 °C in the following primary antibodies diluted in blocking buffer: anti-MAP2 (1:700; Abcam, Cambridge, UK, # Ab5392), SMI312 (1:250; BioLegend, CA, # 837904), anti-HuD (1:400; Abcam, # Ab96474), anti-KHSRP (1:500; Novus, CO, # NBP1-18910), Tuj1 (1:500; Novus, # NB100-1612), anti-Synaptophysin (1:200; Abcam, # Ab32594), anti-GAP43 (1:500; Novus, # NB300-143) and anti-GFP (1:500; Aves, CA, # GFP1020). After washes in PBST, coverslips were incubated for 1 h with combination of FITC-conjugated donkey anti-mouse, Cy5-conjugated donkey anti-chicken, and Cy3-conjugated donkey anti-rabbit antibodies (1:500 each; Jackson ImmunoRes., PA) diluted in blocking buffer. Samples were washed 3 times in PBS, rinsed with distilled H_2_O, and mounted with Prolong Gold Antifade with DAPI (Life Technologies, MA).

For immunostaining tissue sections, brain samples were perfusion fixed in 4 % buffered PFA in PBS, cryoprotected in 30% sucrose overnight, and they cryosectioned at 20-25 µm thickness. Cryosections were stored at −20 °C until used. Sections were thawed to room temperature, washed with PBS, and the permeabilized with 0.3% Triton-X 100. Sections were then blocked with 10% normal donkey serum in PBST (blocking buffer) for 1 h followed by overnight incubation at 4 °C in the following antibodies diluted in blocking buffer: anti-KHSRP (1:500; Novus, NBP1-18910), anti-PSD95 (1:200; Abcam, # Ab2723), and anti-synaptophysin (1:200; Abcam, #Ab32594). After washing 3 times in PBST, sections were incubated 1 h at room temperature with the following secondary conjugated antibodies diluted in blocking buffer: Cy3-conjugated donkey anti-mouse and Cy5-conjugated donkey anti-rabbit (1:500 for both; Jackson ImmunoRes, PA). After 3 washes in PBS, sections were mounted with Prolong Gold with DAPI.

### Immunoblotting

Protein was isolated from cortex dissected from mice were minced and lysed in radioimmunoprecipitation assay (RIPA; Pierce) buffer plus protease inhibitors by passage through a 21 G needle. Samples then vortexed for 3 minutes and rotated 4 °C overnight. Resulting lysed tissue centrifuged at 20,000 × *g* for 10 min at 4 °C. Protein concentrations of supernatants were determined Pierce BCA Protein Assay Kit (ThermoFisher). After normalization for protein concentrations, lysates were denatured in Laemmli sample buffer at 95 °C × 5 min and fractionated by standard SDS/PAGE. Fractionated proteins were electrophoretically transferred to PVDF membranes and blocked in 5% Milk in TBST. Blots were then incubated overnight at 4 °C with the following antibodies diluted in Tris-buffered saline with 0.1% triton 100 plus 5% bovine serum albumin (blocking buffer): anti-KHSRP (1:5000; Novus, #NBP1-18910), anti-FUBP1 (1:2000; Abcam, #Ab181111), anti-SNAP25 (Invitrogen, CA, #PA1-9102; 1:3000), anti-α-Tubulin (1:100; Cell Signaling, MA, # 2125S0), and anti-GAPDH (1:1000; Cell Signaling, #5174). After rinsing in TBST, blots were incubated for 1 h at room temperature in the following HRP-conjugated secondary antibodies diluted in 5% milk in TBST buffer: goat anti-rabbit (1:5000; Cell Signaling, # 7074S) & donkey anti-goat (1:5000; Jackson ImmunoRes, #705-035-003). Blots were then extensively rinsed in TBST and immunocomplexes were visualized using the Clarity ECL Western Blotting Substrate (BioRad) and Biorad ChemiDoc system.

#### Morphological assessments of axon and dendrite growth in KHSRP deficient mice

We used *Khsrp*^*−/−*^ mice crossed with Thy1-GFP mice to analyze axon and dendrite growth in vivo. Adult littermates (2–4-month old) consisting of Thy1-GFP/*Khsrp*^+/+^, Thy1-GFP/*Khsrp*^+/−^ and Thy1-GFP/*Khsrp*^−/−^ were perfused intracardially first with phosphate-buffered saline (PBS; 37 °C), followed by cold 4% paraformaldehyde (PFA, w/v) in PBS. Brains were post-fixed in 4% PFA at 4 °C for 4 h and cryoprotected in 30% sucrose (w/v) in PBS for at least 2 d at 4 °C. 50 μm thick coronal slices were cut on a freezing microtome. Sections were mounted onto coverslips coated with *Vectashield* (Vector Laboratories). For axonal growth, we assessed of the length of hippocampal mossy fiber IPB. Briefly, the length of GFP-positive mossy fibers in the IPB was measured from the cross section of the hilus at the end of the granule cell layer to the point they cross the pyramidal cell layer. IPB length was divided by the total length of the most medial aspect of the hilus to the apex of the curvature of CA3 as previously described^26^.

For analyses of dendrite morphology in the KHSRP knockout mice, confocal images of apical dendrites of layer V pyramidal neurons in prefrontal cortex (labeled throughout the cell body and the dendritic tree with GFP) were by obtained by Leica SP8X confocal microscope using a 63×/NA 1.4 oil immersion objective at 1 μm Z intervals (1024 × 1024 pixel fields; Wetzlar, Germany). Image stacks consisted of 10–50 optical planes. Second-order dendritic shafts in these images were identified at distance of 100–200 μm from the soma were analyzed using *Neurolucida 360* and *Neurolucida Explorer* software (MBF Bioscience, VT). Spine density was assessed and each spine was categorized based on stalk length and head width as thin, stubby and mushroom using the default software parameters^70^. These analyses were done blind to the individual genotypes.

We used the using the *ImageJ Puncta Analyzer plugin* (written by Bary Wark, available upon request, c.eroglu@cellbio.duke.edu) for analyses of synapse density in brain sections as described^71,72^. Multiple studies have used this approach providing supporting as an accurate estimator of synapse number^73–76^, including validation of synapse numbers previously detected ultrastructurally and by electrophysiologically^72,77–79^. For this, 20 μm thick cryosections from 2–4 mo old *Khsrp*^*−/*−^, *Khsrp*^*+/+*^ and *Khsrp*^*fl/fl*^ × *Syn1:Cre* mice were immunostained with anti-synaptophysin and anti-PSD95 as above. 5 μm thick z-stacks (15 sections/stack at 0.33 µm intervals) of the synaptic region in the somatosensory cortex were obtained by Leica SP8X confocal microscope using a 63x/NA 1.4 oil immersion objective and used for maximum projection. 3 animals/genotype and 3 coronal sections/animal were analyzed in layer 4–5 of the somatosensory cortex. *Puncta Analyzer* was used to count the number of pre- and post-synaptic puncta separately and then assessed for puncta colocalizing in close proximity using the same plugin^72^. Merged RGB maximum projections of synaptophysin (red) and PSD95 (green) were background subtracted using rolling ball radius = 50, and then processed through *Puncta Analyzer* for thresholding and puncta colocalizations as described^72^.

#### Analysis of neuronal morphology in culture

Cultured hippocampal and cortical neurons from *Khsrp*^*+/−*^ crosses were assessed for axonal and dendritic growth at DIV7 and for dendritic spine density and morphology at DIV23. DIV7 cultures were fixed in 4% PFA and processed for immunofluorescence with MAP2 and SMI-312 to identify dendrites and axons, respectively. Images for neuronal morphology of DIV 7 neurons were captured on Leica DMI6000 epifluorescent microscope with ORCA Flash ER CCD camera (Hamamatsu Photonics, Shizuoka, Japan) with a 40×/NA 1.2 oil immersion objective using Leica LAS AF as tile scans taken randomly across each coverslip/well. For analyses of dendritic spines, GFP-expressing DIV23 cultures were fixed with 4% PFA and imaged by confocal microscopy using Leica SP8X as above. For this, GFP-filled spines along 20 µm dendrite segments were imaged as Z stacks using 63×/NA 1.4 oil immersion objective. For both DIV7 and DIV23, neurons were imaged blinded to genotype.

Axon and dendrite morphology in cultured neurons was analyzed from epifluorescent tile scan images using *WIS-NeuroMath*^80^ to give average length of each process/neuron and branch density along each axon and dendrite. Dendritic spines in cultured neurons were traced from confocal image stacks using *Neurolucida 360* as above to quantitate spine density and spine type.

For analyses of synapse density in cultured neurons, GFP-expressing DIV23 cultures were immunostained with anti-synaptophysin as above and imaged by confocal microscopy. 30 µm segments of dendrites at least 200 µm from the neuronal cell body were analyzed. GFP-filled spines were marked as ROIs and overlaid with the coordinating synaptophysin channel. The number of synaptophysin labeled puncta that colocalized with the marked ROIs were counted as synapses. Synaptophysin signals within the center of the GFP filled dendrites were excluded from analyses. Dendrite length was also recorded to calculate the average number of synapses per 10 µm. To calculate the % colocalized points, the number of synapses recorded along each dendrite was divided by the total number of GFP filled spines along that dendrite segment.

### Electrophysiological analyses

Coronal slices (300 µm thick) containing either the infralimbic cortex (PFC) or the dorsal hippocampus were prepared from adult mice according to published protocols^81,82^ and maintained in artificial cerebrospinal fluid solution composed of 130 mM NaCl, 3 mM KCl, 1.25 mM NaH_2_PO_4_, 26 mM NaHCO_3_, 10 mM glucose, 1 mM MgCl_2_, and 2 mM CaCl_2_. Slices were continuously perfused with artificial cerebrospinal fluid (aCSF) heated to 32 ± 1 °C. Whole-cell patch clamp recordings were done with a K-gluconate based intracellular solution. mEPSCs were recorded at a holding potential of −65 mV in the presence of bath-applied 1 µM tetrodotoxin. Cells from 3–6 mice were analyzed per genotype. Mean mEPSC amplitude and duration were measured from an average trace of 50–100 individual mEPSCs. Duration was computed as a monoexponential fit to the decay phase of the average mEPSC.

### Behavioral studies

Animals were maintained on a reverse 12 h dark/light cycle (lights on at 20:00 h) in grouped-housed cages. Behavioral testing was conducted using adult male and female *Khsrp*^−/−^, *Khsrp*^+/−^ and *Khsrp*^+/+^ mice that were age- and sex-matched. Note that as KHSRP has also been recently identified to interact with the circadian rhythm by targeting PER2^83^; all behavioral measurements were conducted during the dark period between 09:00 and 17:00 in behavioral rooms lit with red lighting.

Two cohorts of mice consisting of 28 *Khsrp*^*+/+*^ (15 male and 13 female), 33 *Khsrp*^−/−^ (17 male and 16 female), and 10 *Khsrp*^+/−^ (5 male and 5 female) mice were used for behavioral studies as indicated below. Cohort 1 (32 mice) began preliminary behavioral screens between the ages of 9–18 weeks, followed by zero maze at 9–19, open field at 10–21, and ASST at ages 17–27 weeks. Cohort 2 (39 mice) began the preliminary behavioral screen between the ages of 10–21 weeks, followed by zero maze at 10–24, open field at 11–24, ASST at 18–29 weeks, and trace fear conditioning at 23–33 weeks.

All mice were assessed in a preliminary behavioral screen using a subset of tests derived from the Irwin screen as previously described^30–32^ for physical health, sensory, motor, and neurological function. Exploratory behaviors were observed by placing the mouse in a corner of a clear box (45 × 45 × 22 cm) and recording for 10 min.

Elevated zero-maze test was conducted as previously described^84^ on a white circular platform (5 cm runway, 60 cm diameter and 50 cm from the floor) consisting of 2 opposing open quadrants with a 0.5 cm raised lip to prevent falling and 2 opposing closed quadrants with 15 cm high walls. The room was illuminated with red fluorescent lights and two single white lights (open arms 90 lx, closed arms 45 lux). Mice were allowed to freely explore the arena for 5 min. Locomotor activity and time spent in the open vs closed arms was measured using *Ethovision* video tracking system (Noldus Information Technology, VA).

Open field test was conducted as previously described^85^ in a square arena (40 × 40 × 35 cm) constructed from white Plexiglas. The room was illuminated with red fluorescent lights and two single white lights (center 60 lx, corner 35 lx). Mice were placed in the NW corner of the arena and allowed to freely explore for 30 min per day for 5 consecutive days to establish a baseline of anxiety and locomotor activity. Total distance traveled, velocity, and duration in the (20 × 20 cm) center was measured using *Ethovision*.

ASST was conducted as previously described^47,86,87^. Testing was conducted in an acrylic apparatus (30 × 18 × 12 cm) divided into a start box and 2 choice chambers. Each choice chamber contained a ceramic digging bowl (4.5 × 2.5 cm) placed on an in house manufactured platform (11 × 5 cm) with sandpaper, wood, neoprene, metal wire, tile, or a plastic fiber sponge as textures. Scented medium was made by mixing 150 g of cob bedding with 20 crushed 14 mg dustless precision pellets (#F0568, BioServ, NJ) and 3 g of commercially available powdered spices: nutmeg, ginger, garlic, coriander, thyme, and cinnamon (Kroger Co., OH). Prior to training animals were reduced to 85% free feeding weight and acclimated to food reward in the home cage. Training day 1 consisted of acclimation to the testing chamber and digging in unscented cob medium for reward. Training day 2 introduced the mice to all exemplar combinations encountered during testing (Supplementary Fig. 8a). A single pellet was placed below the cob in one bowl, randomly assigned between trials, and placement was mimicked in the unrewarded bowl to prevent mice learning experimenter cues. Testing on day 3 was conducted in succession with no inter-session-breaks on 7 discrimination tasks (Supplementary Fig. 8a). In simple discrimination (SD) mice were trained to discriminate 2 exemplars in either the odor or platform dimension (counterbalanced across genotypes and sex *n* = 34). Upon reaching criterion, mice were moved to compound discrimination (CD) in which the second, non-rewarded dimension, was added. The rewarded exemplar in the initially rewarded dimension was then reversed to form a compound discrimination reversal (CDR). Next, a novel set of exemplars in each dimension were introduced and mice were rewarded for responding to one exemplar in the initially learned dimension (intra-dimensional shift [IDS]). Next, the intra-dimensional reversal (IDR) reversed the correct stimuli within the same dimension. A second novel set of exemplars in both dimensions were introduced in the extra-dimensional shift (EDS) where the rewarded exemplar was in the previously irrelevant dimension. Finally, the correct exemplar within the newly learned dimension was reversed to form an extra-dimensional reversal (EDR).

Trials to criterion, corrects, and errors were recorded for each stage. If a mouse did not dig in either bowl by 2 min the trial was recorded as ‘no choice’ and the mouse repeated the trial until a choice was made. Criterion was set to 6 consecutive correct responses. Trial latencies to respond were measured from the time the barrier was raised until digging was initiated. A dig was defined as the moment when the mouse’s nose or paw broke the surface of the cob-digging medium. Mice were discontinued if they required 60 trials on any 1 problem or 150 trials total.

Trace fear conditioning studies were conducted between 0900 and 1200 h under dim red illumination, as previously described^88^. Briefly, animals were placed into a Coulburn Instruments (Whitehall, PA) Habitest System for 90 s of habituation, followed by 7 trials each consisting of the CS (10 s, 80 dB 6 Hz clicker), a 30 s trace, the US (1 s, 0.8 mA scrambled foot shock), and a 180 s inter-trial interval. The subject was removed from the chamber 60 s following the delivery of the last US. 24 h later, freezing to the CS in a novel context (a standard, clean mouse cage with minimal bedding) was assessed. The CS was delivered at 180, 310, and 440 s. The animal’s behavior was videotaped, and the amount of time spent freezing during the tone and during the 30 s trace (40 s total duration) was scored for each CS by 2 investigators, one of which was blinded to the genotype. The average for the 3 measures was calculated and expressed as a percentage of time spent freezing.

### Statistical Analyses

All statistical tests were performed using *Prism* software package (version 8.4.0; GraphPad). One-way ANOVAs were used to compare the molecular, morphological, and electrophysiological differences in *Khsrp*^+/−^, *Khsrp*^−/−^ and *Khsrp*^+/+^ mice and post-hoc Tukey tests were used to identify significant changes between two genotypes. Student *t*-tests were used for the analyses of KHSRP-target mRNAs levels in *Khsrp*^−/−^ and *Khsrp*^*+/−*^ vs. *Khsrp*^+/+^ mice. Repeated measures (RM) ANOVA were used for analyses of percent center duration, distance and velocity d 1–5, distances and velocity d 1, 0–5 min through 20-25 in the open field. Tukey post-hoc tests were utilized for open field distance and velocity d 1–5, distances and velocity d 1, 0–5 through 20–25 min. Two-way ANOVAs were used to determine if there was a main effect of sex and genotype for the percent open arm duration, distance, and velocity in the zero maze. RM ANOVA was used to determine overall effects of problem stages in ASST and one-way ANOVA was used to compare starting dimension effects of specific stages. To compare each genotype between stages (CDR-CD, IDR-IDS, and EDR-IDS) Student *t*-tests were employed. RM ANOVA was utilized for the Tone Training, Trace Training, as well as the Tone-Trace Test followed by individual one-way ANOVAs for each time point in Trace Fear Conditioning.

### Reporting summary

Further information on research design is available in the Nature Research Reporting Summary linked to this article.

## Supplementary information

Supplementary Information

Description of Additional Supplementary Files

Supplementary Data 1-9

Supplementary Data 10

Supplementary Data 11

Supplementary Data 12

Supplementary Data 13

Supplementary Data 14

Supplementary Data 15

Supplementary Data 16

Supplementary Data 17

Supplementary Data 18

Supplementary Data 19

Supplementary Data 21

Supplementary Data 20

Supplementary Data 22

Supplementary Data 23

Reporting Summary

## Data Availability

The data sets for cDNA arrays and RIP-Seq are available in the NCBI GEO repository (accession number = GSE166010). Excel files for initial analyses of cDNA array and RIP-Seq, integration of array and RIP-Seq data and analyses of ARE sites in KHSRP-target mRNAs are provided as Supplementary Data 1–4. RNA copy number from RTddPCr analyses are provided as Supplementary Data 5–8. Primer sets used for genotyping and RTddPCR are provided as Supplementary Data 9. Excel files for source data for analyses here are provided as Supplementary Data 10–23.
