# Supplementary material for: KHSRP loss increases neuronal growth and synaptic transmission and alters memory consolidation through RNA stabilization
Source: Commun Biol. 2022 Jul 7;5:672. doi: 10.1038/s42003-022-03594-4 (PMC9262970; doi:10.1038/s42003-022-03594-4)
Supplement: Supplementary file 1 — Supplementary Information [file 42003_2022_3594_MOESM1_ESM.pdf]

SUPPLEMENTARY FIGURES

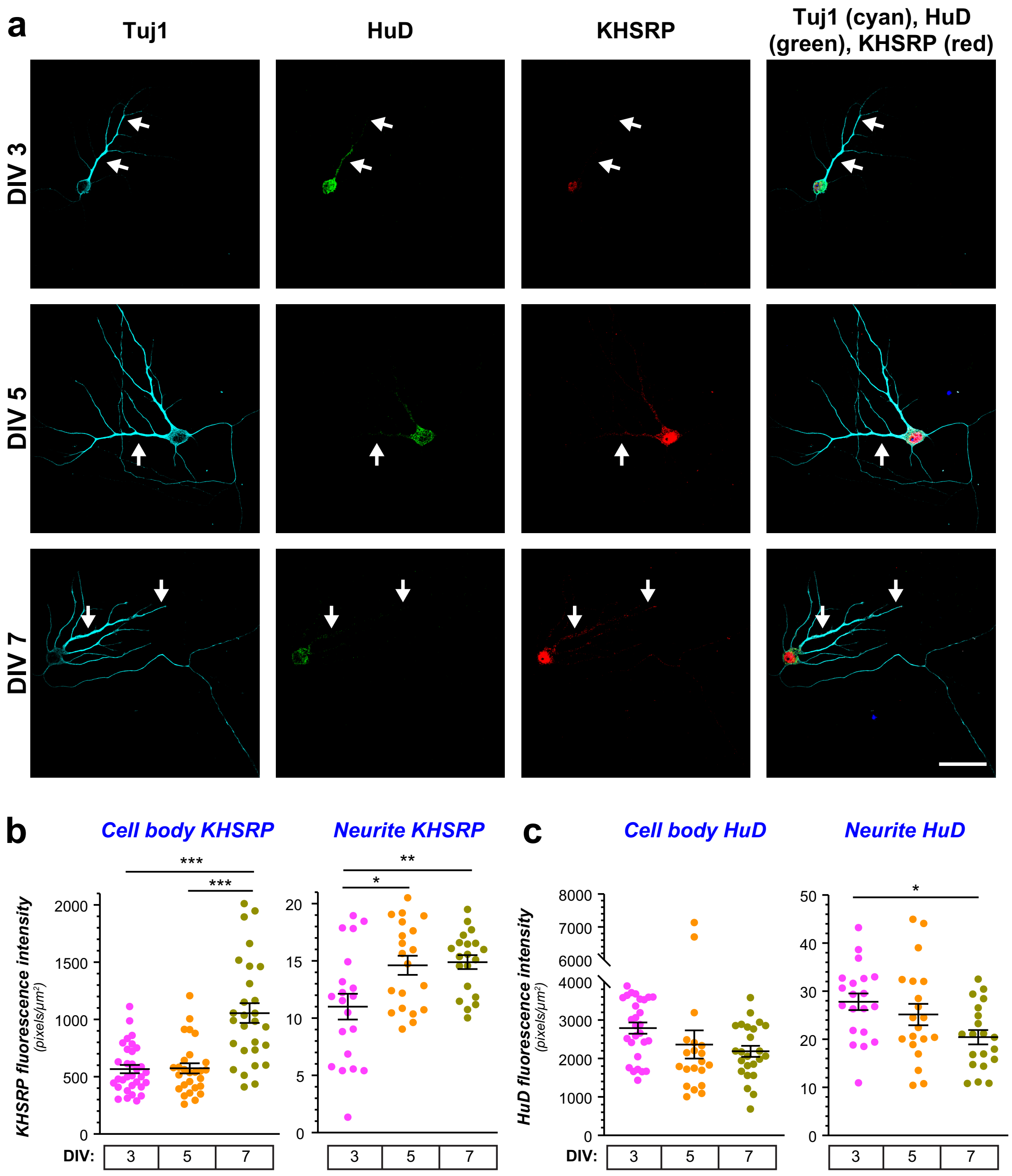

Supplementary Figure 1: ***KHSRP expression progressively increases with maturation of neuronal cultures.***

a) Representative epifluorescent images for Tuj1 (cyan), HuD (green) and KHSRP (red) for DIV3-7 neocortical neuron cultures from wild type mice are shown [scale bar = 100 µm]. Imaging parameters for individual channels are matched for exposure and gain across each respective column. Arrows indicate neurites where there is a progressive increase in KHSRP and decrease in HuD over days in culture.

b-c) Quantification of signal intensities from cultures as in a for KHSRP (**b**) and HuD (**c**) in cell bodies and neurites is shown (N > 30 neurons in at least 3 separate cultures; * $p \leq 0.05$, ** $p \leq 0.01$, and *** $p \leq 0.005$ by one-way ANOVA with Tukey post-hoc compared to DIV3 as indicated).

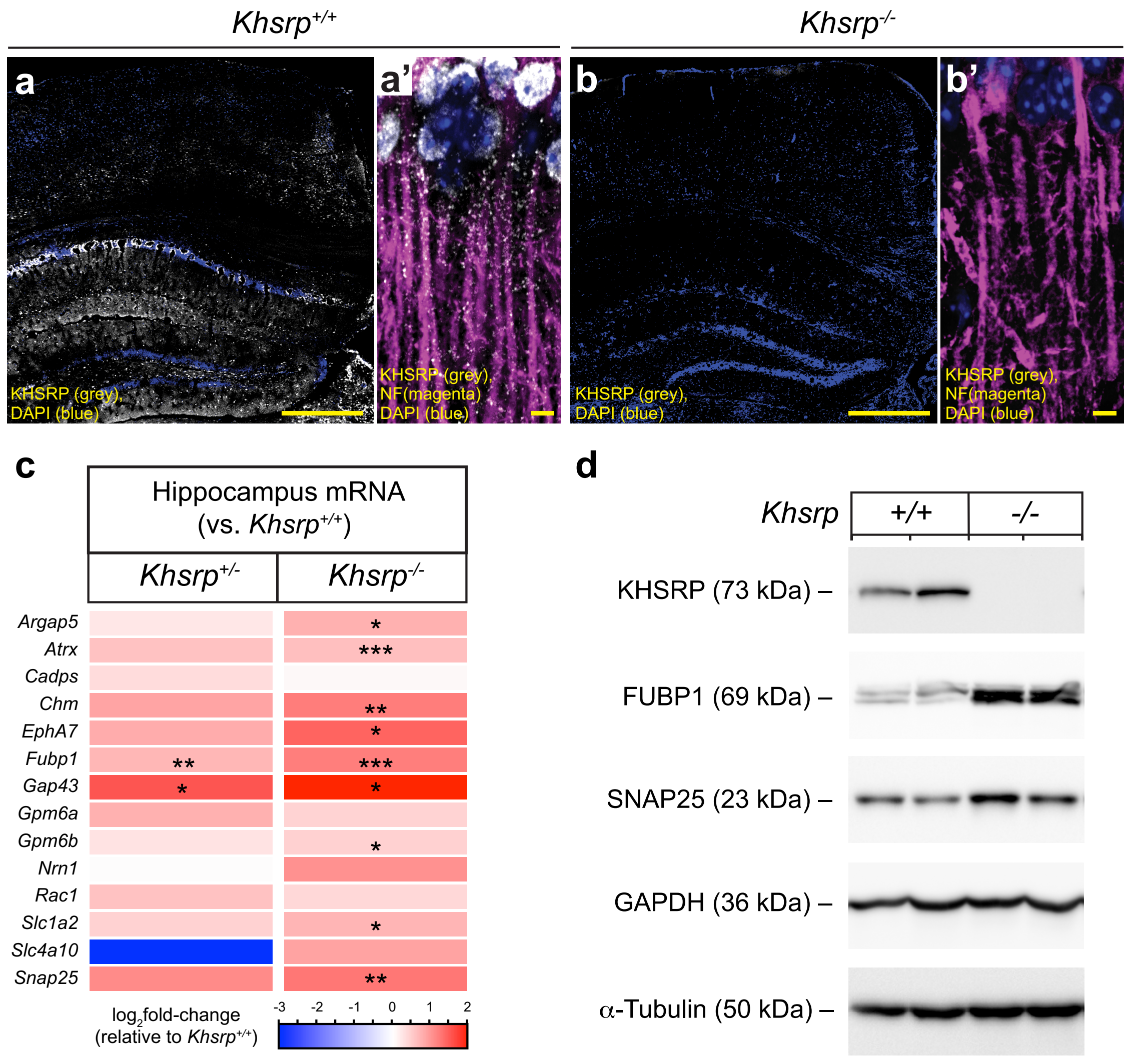

<u>**Supplementary Figure 2**</u>: ***Elevated KHSRP-target mRNAs and proteins in KHSRP deficient mice.***

a-b) Representative images from anti-KHSRP immunofluorescent staining of brain sections of *Khsrp*$^{+/+}$ (**a**) and *Khsrp*$^{-/-}$ (**b**) adult mice. The boxed regions in panel **a** are represented at higher power in panels **a'** and **a''** for somatosensory cortex and hippocampus, respectively.

c) Heatmap for $\log_2$ fold-change for KHSRP-target mRNAs in *Khsrp*$^{+/-}$ and *Khsrp*$^{-/-}$ vs. *Khsrp*$^{+/+}$ hippocampal RNA isolations is shown (N ≥ 4 per condition; * $p \leq 0.05$, ** $p \leq 0.01$, and *** $p \leq 0.005$ by two-tailed Student's *t*-test).

d) Representative immunoblots from lysates of cortex of adult *Khsrp*$^{+/+}$ vs. *Khsrp*$^{-/-}$ mice are shown for 2 littermates of each genotype. The loss of KHSRP seen by immunoblotting is accompanied by increased levels of SNAP25 and FUBP1 proteins. GAPDH and α-Tubulin signals show approximately equal loading between the lanes.

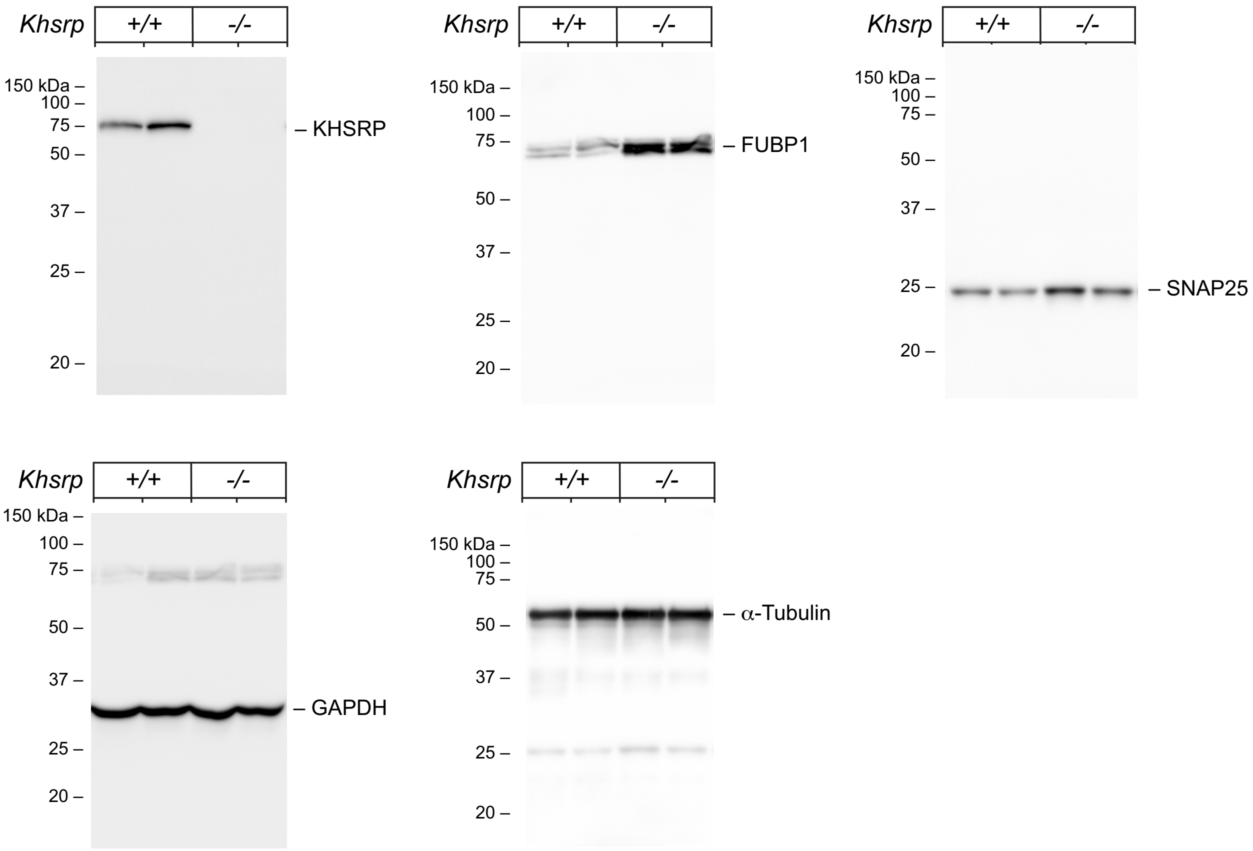

<u>Supplementary Figure 3</u>: ***Increased SNAP25 and FUBP1 proteins in KHSRP deficient mice.***
Uncropped blots corresponding to data shown in Supplementary Figure 2d.

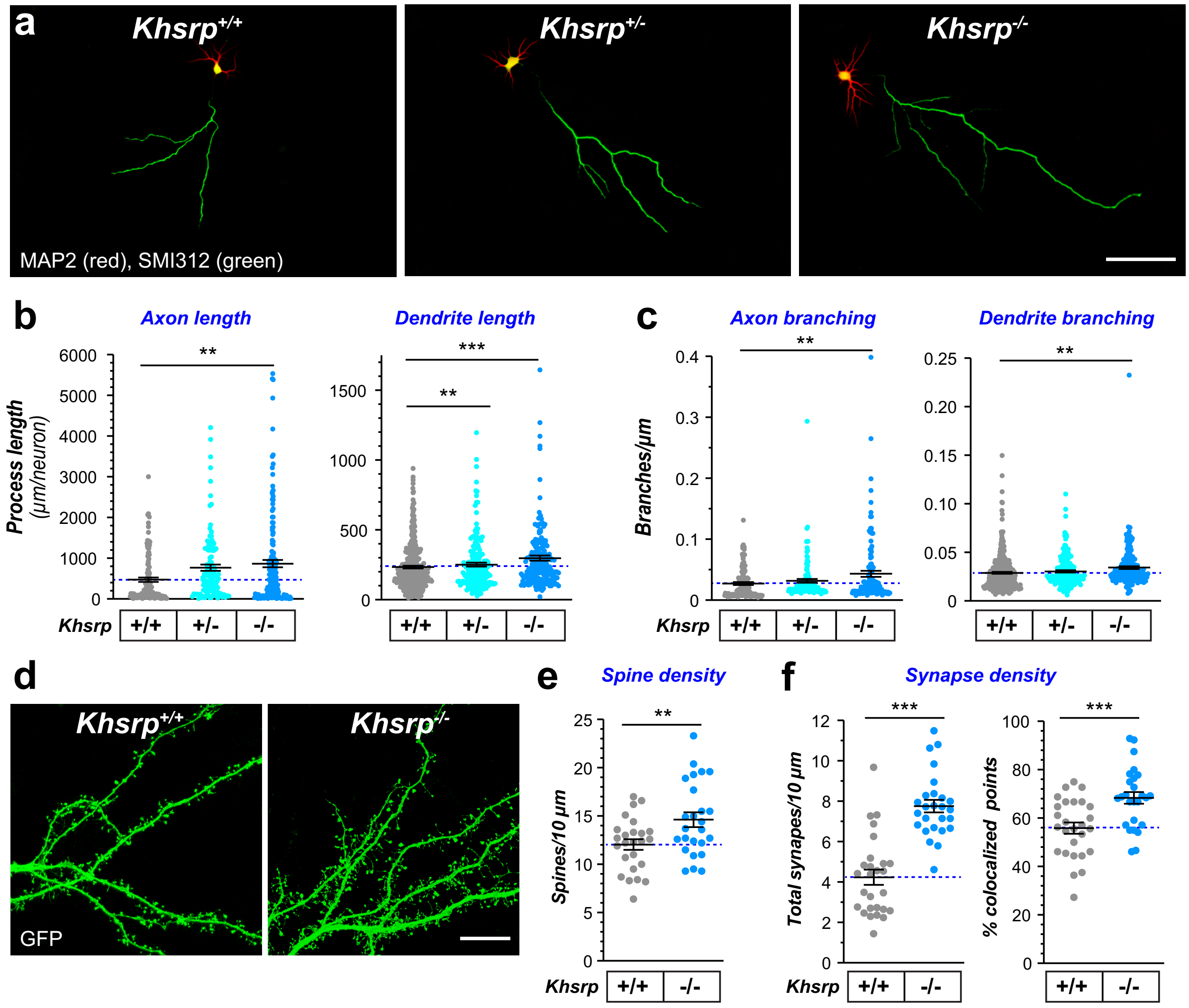

Supplementary Figure 4: ***KHSRP deficient hippocampal neurons show increased axon and dendrite growth and synaptogenesis.***

a) Representative epifluorescent images for MAP2 (red) and SMI312 (green) immunostained hippocampal neuron cultures at DIV7 from *Khsrp*$^{+/+}$, *Khsrp*$^{+/-}$, and *Khsrp*$^{-/-}$ mice.

b-c) Quantification of length (**b**) and branching (**c**) for axons and dendrites from DIV7 hippocampal cultures from *Khsrp*$^{+/+}$, *Khsrp*$^{+/-}$ and *Khsrp*$^{-/-}$ mice (N > 30 neurons in at least 3 separate cultures; ** $p \leq 0.01$ and *** $p \leq 0.005$ by one-way ANOVA with Tukey post-hoc compared to DIV3 as indicated).

d-f) Representative epifluorescent images of distal dendrites for DIV23 hippocampal neurons (**d**), dendrite spine density (**e**), and synapse density (**f**) for *Khsrp*$^{+/+}$ vs. *Khsrp*$^{-/-}$ mice shown as indicated (N > 30 neurons in at least 3 separate cultures; * $p \leq 0.05$ and ** $p \leq 0.01$ by Student's *t*-test).

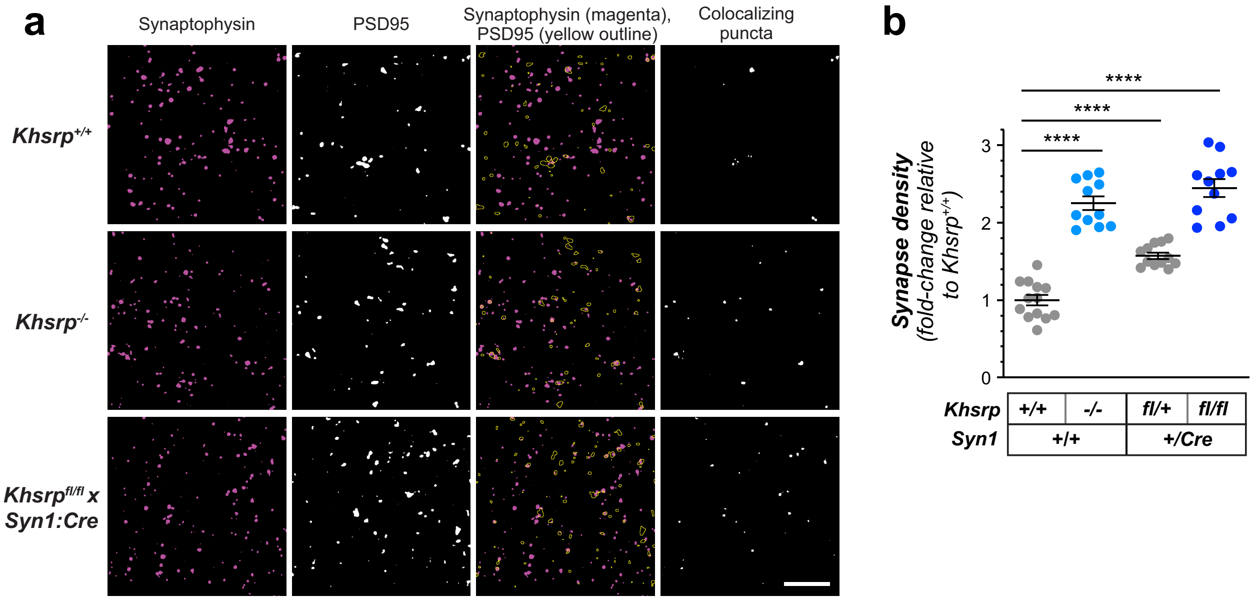

Supplementary Figure 5: Both constitutive and neuron-specific *Khsrp* knockout mice show increased synapse density *in vivo*.

a) Representative confocal images for immunostained somatosensory cortex used to determine synapse density based on pre-synaptic (synaptophysin) and post-synaptic (PSD95) marker colocalization in close proximity using *Puncta Analyzer* in *ImageJ*. Images for *Khsrp*$^{+/+}$, *Khsrp*$^{-/-}$, *Khsrp*$^{fl/fl}$ *x Syn1:Cre* mice shown as indicated. Third column shows merge of pre- and post-synaptic protein signals from *Puncta Analyzer* with the post-synaptic signals outlined in yellow. Fourth column shows synaptic puncta where synaptophysin and PSD95 signals are in close proximity as defined by *Puncta Analyzer* [scale bar = 5 µm].
b) Quantitation of the synaptic puncta from images as in a is shown for *Khsrp*$^{+/+}$, *Khsrp*$^{-/-}$, *Khsrp*$^{+/fl}$ *x Syn1:Cre, and Khsrp*$^{fl/fl}$ *x Syn1:Cre* mice is shown. In each case, loss of KHSRP increases synapse numbers (N=4 Z-stacks/animal, 3 animals/genotype; *** p ≤ 0.005 and *** p<0.001 by one-way ANOVA).

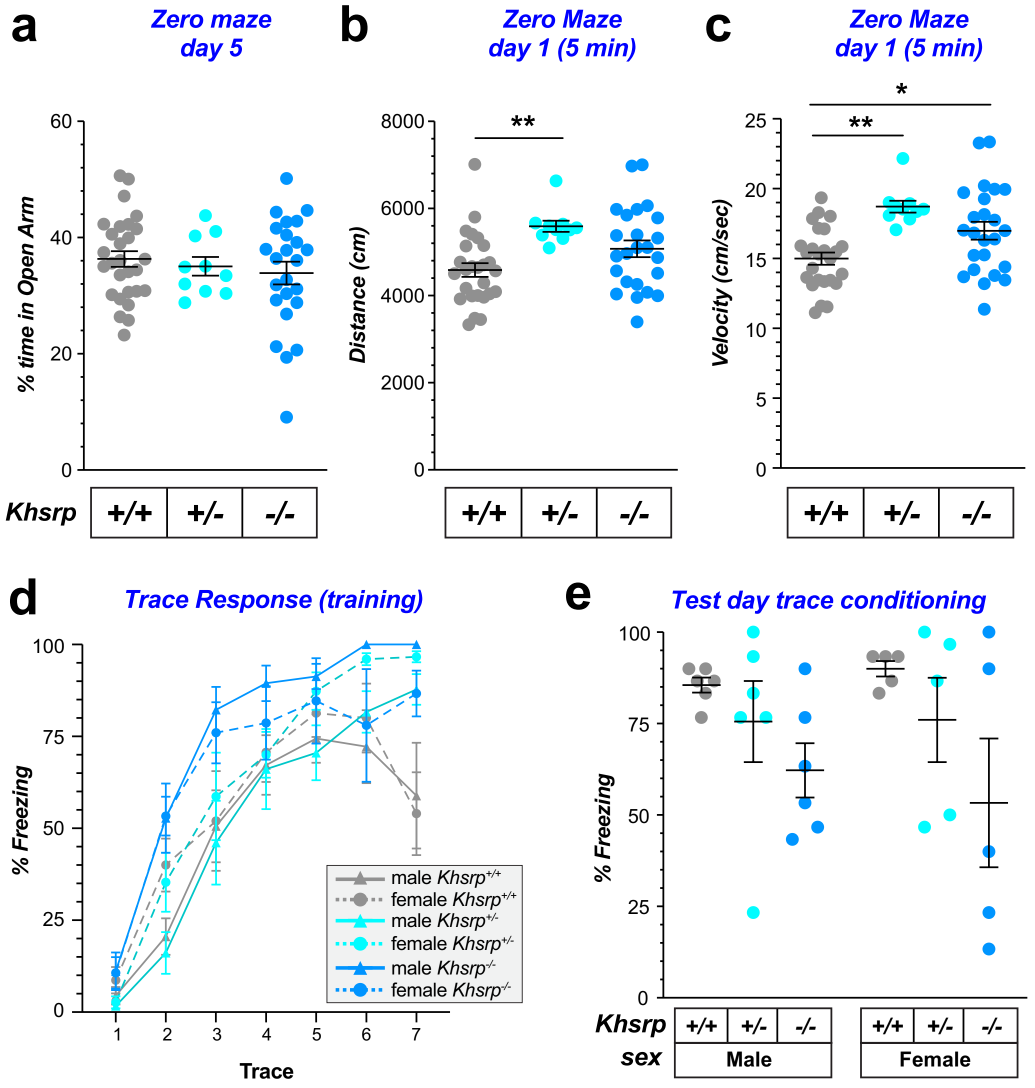

<u>Supplementary Figure 6</u>: ***Increased locomotor activity, but no sex differences for KHSRP deficient mice.***

a-c) Adult *Khsrp*$^{+/+}$ (n=28), *Khsrp*$^{+/-}$ (n=10) and *Khsrp*$^{-/-}$ (n=25) of both sexes were placed in a Zero Maze apparatus and the percentage time in the open arm (**a**), distance travelled (**b**) and velocity (**c**) were determined both at day 1 and day 5 of training. No changes were observed on percentage time in the open arm, but ANOVA analyses showed increased locomotor activity as measured by distance traveled and velocity in the zero maze (ANOVA for distance traveled: F(2,52)=4.606 p=0.0143, and for velocity: F(2,53)=6.915, p=0.0022; *p<0.05 and **p<0.01 using Dunnett's multiple comparisons test).

d-e) The freezing during training (**d**) or testing (**e**) did not differ significantly in males vs. female mice (n=5 for male mice and n=4 for female mice of all genotypes; no significant differences in a by repeated measures ANOVAs and in b by One-way ANOVAs for each sex).

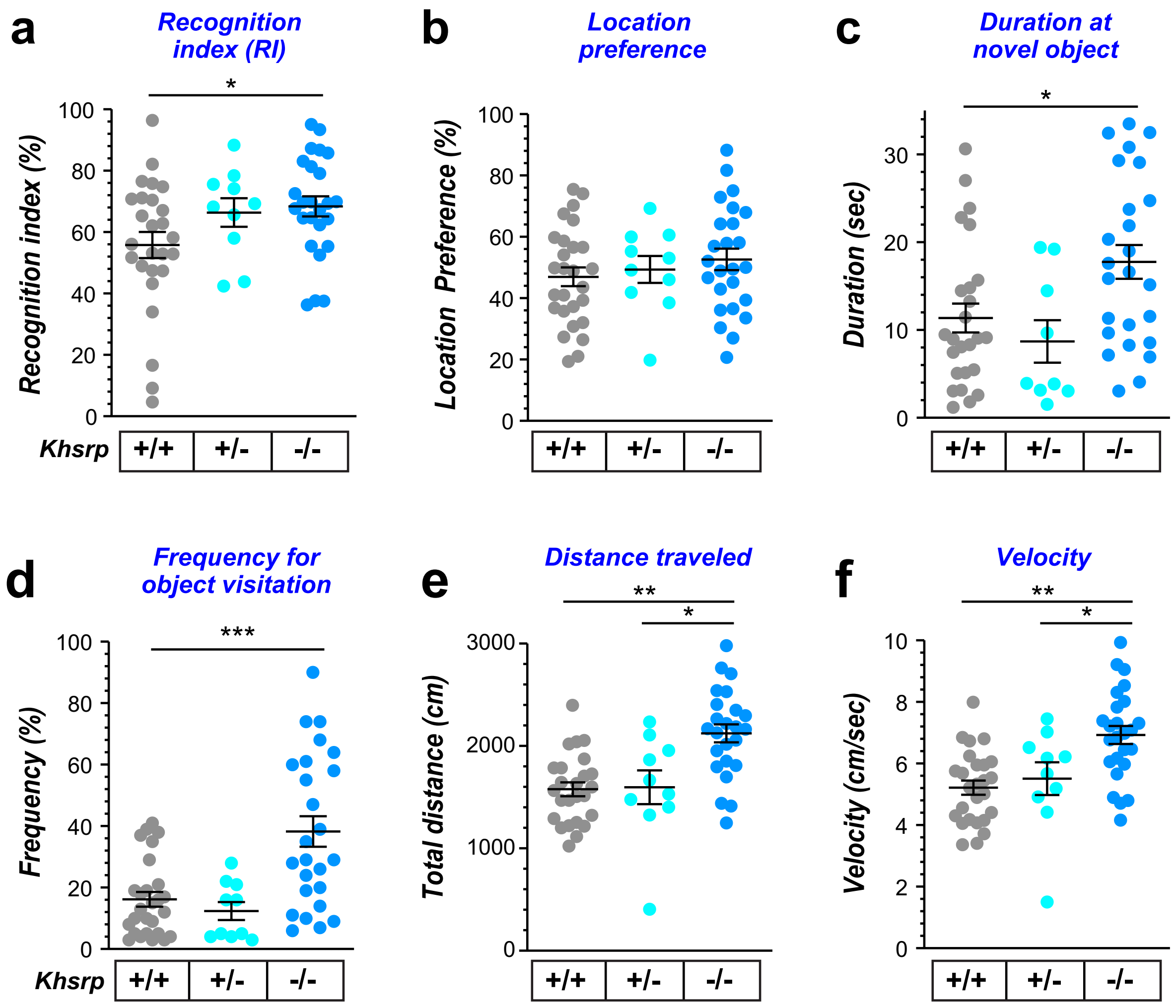

<u>Supplementary Figure 7</u>: *KHSRP deficient mice display increased novel object recognition.*

a) *Khsrp*$^{-/-}$ mice have an increased recognition index during the test phase of novel object recognition.

b) None of the mice exhibited location preference of sample objects, dotted line represents 50%.

c) *Khsrp*$^{-/-}$ mice spend more time within 1cm of the novel object.

d) *Khsrp*$^{-/-}$ mice also have increased visits to the novel object.

e-f) Locomotor activity was increased in *Khsrp*$^{-/-}$ mice during the test phase for both total distance traveled (**e**) and velocity (**f**). Data are displayed mean ± SEM (*Khsrp*$^{+/+}$ n= 15 males and 13 females; *Khsrp*$^{+/-}$ n=5 males and 5 females; and *Khsrp*$^{-/-}$ n=12 males and 13 females; * p≤0.05, ** p≤0.01, *** p≤0.001 by Welch's t-tests).

a

Stage	Odor as starting dimension (blue font = rewarded)	
**Simple discrimination (SD)**<br>Learn which odor or platform is rewarded.	**Nutmeg**	Ginger
**Compound discrimination (CD)**<br>Add relevant dimension (i.e., platform for odor starting dimension), keep rewarded contingency the same as in previous stage.	**Ginger** / Sandpaper<br>**Ginger** / Wood	Nutmeg / Wood<br>Nutmeg / Sandpaper
**Intra-dimensional shift (IDS)**<br>Introduce new exemplars for discrimination and keep the rewarded dimension the same as in previous stages (i.e., odor to odor).	**Ginger** / Neoprene<br>**Garlic** / Metal	Coriander / Metal<br>Coriander / Neoprene
**Intra-dimensional shift reversal (IDR)**<br>Reverse which odor or platform is rewarded from the same dimension as in previous stages.	**Coriander** / Neoprene<br>**Coriander** / Metal	Garlic / Metal<br>Garlic / Neoprene
**Extra-dimensional shift (EDS)**<br>Introduce new exemplars for discrimination and change to the irrelevant dimension from prevous stage (i.e., odor to platform).	Thyme / **Tile**<br>Thyme / Scrubber	Cinnamon / Scrubber<br>Cinnamon / **Tile**
**Extra-dimensional shift reversal (EDR)**<br>Reverse which odor or platform is rewarded from the same dimension as previous stage.	Cinnamon / Tile<br>Cinnamon / **Scrubber**	Thyme / **Scrubber**<br>Thyme / Tile

b

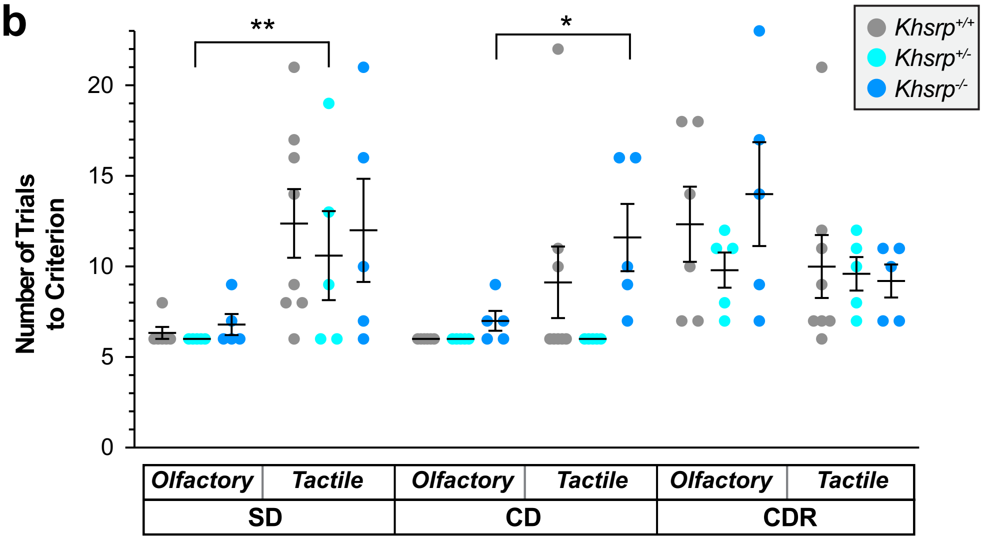

Supplementary Figure 8: ***Odor and platform cues for the Attentional set shifting task (ASST).***
a) Summary of cues used for the ASST stages, with odor as the starting dimension.
b) There is a significant main effect of starting dimension during the Simple Discrimination (SD) and Compound Discrimination (CD) stages that was eliminated by the Compound Discrimination Reversal (CDR) stage. Tactile learning is more difficult to initially acquire than olfactory learning[47], but mice of the three genotypes learn to efficiently discriminate tactile differences after repeated exposures (Figure 7B) (N ≥ 10 mice/genotype; *p<0.05 and **p<0.01 by two-way ANOVA, tactile vs. olfactory for SD [F (1, 28) = 13.00 P=0.0012], CD [F (1, 28) = 5.205 P=0.0303], and CDR [F (1, 28) = 2.672 P=0.1133]).
