# Supplementary material for: KHSRP loss increases neuronal growth and synaptic transmission and alters memory consolidation through RNA stabilization
Source: Commun Biol. 2022 Jul 7;5:672. doi: 10.1038/s42003-022-03594-4 (PMC9262970; doi:10.1038/s42003-022-03594-4)
Supplement: Supplementary file 2 — Description of Additional Supplementary Files [file 42003_2022_3594_MOESM2_ESM.pdf]

DESCRIPTION OF ADDITIONAL SUPPLEMENTARY FILES

Supplementary Data 1: *Analyses of mRNA levels in KHSRP deficient mouse neocortex.* DNA microarray data for Khsrp$^{-/-}$ vs. Khspr$^{+/+}$ mice.

Supplementary Data 2: *Identification of KHSRP-target mRNAs through RIP-Seq*. RIP-seq data for KHSRP from wild type KHSRP mouse neocortex.

Supplementary Data 3: *Overlapping datasets of KHSRP targets.* Integration of data from Supplementary Data 1 and 2, showing mRNAs that are increased in *Khsrp*$^{-/-}$ vs. *Khsrp*$^{+/+}$ neocortex that also showed co-immunoprecipitation with KHSRP by RIP-Seq analyses.

Supplementary Data 4: *KHSRP-target mRNAs from integrated expression and RIP-Seq data contain AREs.* Summary of mRNAs from Supplementary Data 3 that contained AU-rich elements (ARE) in their 3'UTRs.

Supplementary Data 5: *RTddPCR validation of KHSRP-target mRNAs in neocortex*.

Supplementary Data 6: *RTddPCR validation of KHSRP-target mRNAs in hippocampus.*

Supplementary Data 7: *RTddPCR validation of KHSRP-target mRNAs in cortical neuron cultures.*

Supplementary Data 8: *Behavioral analyses of KHSRP deficient mice.*

Supplementary Data 9: *Primers for RTddPCR analyses.*

Supplementary Data 10: Source data for Figure 1e.

Supplementary Data 11: Source data for Figure 2e-g.

Supplementary Data 12: Source data for Figure 3b-d.

Supplementary Data 13: Source data for Figure 4b and d-h.

Supplementary Data 14: Source data for Figure 5b and d.

Supplementary Data 15: Source data for Figure 6a-f.

Supplementary Data 16: Source data for Figure 7b.

Supplementary Data 17: Source data for Supplementary Figure 1b-c.

Supplementary Data 18: Soucre data for Supplementary Figure 2c.

Supplementary Data 19: Source data for Supplementary Figure 4b-f.

Supplementary Data 20: Source data for Supplementary Figure 5b.

Supplementary Data 21: Source data for Supplementary Figure 6a-e.

Supplementary Data 22: Source data for Supplementary Figure 7a-f.

Supplementary Data 23: Source data for Supplementary Figure 8b.
