# Supplementary material for: KHSRP loss increases neuronal growth and synaptic transmission and alters memory consolidation through RNA stabilization
Source: Commun Biol. 2022 Jul 7;5:672. doi: 10.1038/s42003-022-03594-4 (PMC9262970; doi:10.1038/s42003-022-03594-4)
Supplement: Supplementary file 18 — Reporting Summary [file 42003_2022_3594_MOESM18_ESM.pdf]

nature portfolio

Corresponding author(s): Jeffery L Twiss

Last updated by author(s): May 22, 2022

Reporting Summary

Nature Portfolio wishes to improve the reproducibility of the work that we publish. This form provides structure for consistency and transparency in reporting. For further information on Nature Portfolio policies, see our Editorial Policies and the Editorial Policy Checklist.

Statistics

For all statistical analyses, confirm that the following items are present in the figure legend, table legend, main text, or Methods section.

n/a	Confirmed	
☐	☒	The exact sample size (*n*) for each experimental group/condition, given as a discrete number and unit of measurement
☒	☐	A statement on whether measurements were taken from distinct samples or whether the same sample was measured repeatedly
☐	☒	The statistical test(s) used AND whether they are one- or two-sided<br>*Only common tests should be described solely by name; describe more complex techniques in the Methods section.*
☐	☒	A description of all covariates tested
☐	☒	A description of any assumptions or corrections, such as tests of normality and adjustment for multiple comparisons
☐	☒	A full description of the statistical parameters including central tendency (e.g. means) or other basic estimates (e.g. regression coefficient) AND variation (e.g. standard deviation) or associated estimates of uncertainty (e.g. confidence intervals)
☐	☒	For null hypothesis testing, the test statistic (e.g. *F*, *t*, *r*) with confidence intervals, effect sizes, degrees of freedom and *P* value noted<br>*Give P values as exact values whenever suitable.*
☒	☐	For Bayesian analysis, information on the choice of priors and Markov chain Monte Carlo settings
☒	☐	For hierarchical and complex designs, identification of the appropriate level for tests and full reporting of outcomes
☒	☐	Estimates of effect sizes (e.g. Cohen's *d*, Pearson's *r*), indicating how they were calculated

Our web collection on statistics for biologists contains articles on many of the points above.

Software and code

Policy information about availability of computer code

Data collection	N/A
Data analysis	*Provide a description of all commercial, open source and custom code used to analyse the data in this study, specifying the version used OR state that no software was used.*

For manuscripts utilizing custom algorithms or software that are central to the research but not yet described in published literature, software must be made available to editors and reviewers. We strongly encourage code deposition in a community repository (e.g. GitHub). See the Nature Portfolio guidelines for submitting code & software for further information.

Data

Policy information about availability of data

All manuscripts must include a data availability statement. This statement should provide the following information, where applicable:

- Accession codes, unique identifiers, or web links for publicly available datasets
- A description of any restrictions on data availability
- For clinical datasets or third party data, please ensure that the statement adheres to our policy

Provide your data availability statement here.

Field-specific reporting

Please select the one below that is the best fit for your research. If you are not sure, read the appropriate sections before making your selection.

☒ Life sciences ☐ Behavioural & social sciences ☐ Ecological, evolutionary & environmental sciences

For a reference copy of the document with all sections, see nature.com/documents/nr-reporting-summary-flat.pdf

Life sciences study design

All studies must disclose on these points even when the disclosure is negative.

Sample size	Sample size for animal studies using behavioral, microscopic, and electrophysiology analyses was determined power analyses. For microscopic analyses of cultured neurons, sample size was driven by ensuring that all analyses had ≥ 3 experimental replicates and ≥ technical replicates in each condition/genotype.
Data exclusions	no data were excluded from analyses presented here.
Replication	All studies were replicated on multiple animals or at least 3 replicate cell isolations as indicated in methods and figure legends.
Randomization	Samples were allocated into experimental groups based on genotype, with genotype blinded to the experimenter.
Blinding	All experiments were performed blinded whenever feasible.

Reporting for specific materials, systems and methods

We require information from authors about some types of materials, experimental systems and methods used in many studies. Here, indicate whether each material, system or method listed is relevant to your study. If you are not sure if a list item applies to your research, read the appropriate section before selecting a response.

Materials & experimental systems

n/a	Involved in the study	
☐	☒	Antibodies
☒	☐	Eukaryotic cell lines
☒	☐	Palaeontology and archaeology
☐	☒	Animals and other organisms
☒	☐	Human research participants
☒	☐	Clinical data
☒	☐	Dual use research of concern

Methods

n/a	Involved in the study	
☒	☐	ChIP-seq
☒	☐	Flow cytometry
☒	☐	MRI-based neuroimaging

Antibodies

Antibodies used	For immunofluorescence: anti-MAP2 (1:700; Abcam, Cambridge, UK, # Ab5392), SMI312 (1:250; BioLegend, CA, # 837904), anti-HuD (1:400; Abcam, # Ab96474), anti-KHSRP (1:500; Novus, CO, # NBP1-18910), Tuj1 (1:500; Novus, # NB100-1612), anti-Synaptophysin (1:200; Abcam, # Ab32594), anti-GAP43 (1:500; Novus, # NB300-143), anti-PSD95 (1:200; Abcam, # Ab2723), anti-synaptophysin (1:200; Abcam, # Ab32594), and anti-GFP (1:500; Aves, CA, # GFP1020). For Immunoblotting: anti-KHSRP (1:5000; Novus, # NBP1-18910), anti-FUBP1 (1:2000; Abcam, # Ab181111), anti-SNAP25 (Invitrogen, CA, # PA1-9102; 1:3000), anti-α-Tubulin (1:100; Cell Signaling, MA, # 2125S0), and anti-GAPDH (1:1000; Cell Signaling, # 5174). For immunoprecipitation: anti-KHSRP (55 μg/mg lysate; Novus, CO, # NBP1-18910).
Validation	All primary antibodies were validated by western blotting and/or immunofluorescence on knockout mouse tissues

Animals and other organisms

Policy information about studies involving animals; ARRIVE guidelines recommended for reporting animal research

Laboratory animals	C57Bl/6, B6.Cg-Tg(Thy1-EGFP)OJrs/GfngJ, and B6.Cg-Tg(Syn1-cre)671Jxm/J were obtained from Jackson Laboratories; Khsrp-/- mice have deletion of exons 1-13 as described in Lin et al. (2011, Molec. Cell. Biol. 31:3196); C57Bl/6 mice with loxP sites in the Khsrp allele were generated by Biocytogen (Wakefield, MA) using CRISPR/EGE™-based gene editing.
Wild animals	N/A
Field-collected samples	N/A

Ethics oversight	Institutional animal use and care committees from University of South Carolina and University of New Mexico approved all animal studies in this work.

Note that full information on the approval of the study protocol must also be provided in the manuscript.
